# Cost-Effectiveness of Treating Multidrug-Resistant Tuberculosis

**DOI:** 10.1371/journal.pmed.0030241

**Published:** 2006-07-04

**Authors:** Stephen C Resch, Joshua A Salomon, Megan Murray, Milton C Weinstein

**Affiliations:** **1**Department of Health Policy and Management, Harvard School of Public Health, Harvard University, Boston, Massachusetts, United States of America; **2**Department of Population and International Health, Harvard School of Public Health, Harvard University, Boston, Massachusetts, United States of America; **3**Harvard Initiative for Global Health, Harvard University, Cambridge, Massachusetts, United States of America; **4**Department of Epidemiology, Harvard School of Public Health, Harvard University, Boston, Massachusetts, United States of America; **5**Division of Social Medicine and Health Inequalities, Brigham and Women's Hospital, Boston, Massachusetts, United States of America; Emory UniversityUnited States of America

## Abstract

**Background:**

Despite the existence of effective drug treatments, tuberculosis (TB) causes 2 million deaths annually worldwide. Effective treatment is complicated by multidrug-resistant TB (MDR TB) strains that respond only to second-line drugs. We projected the health benefits and cost-effectiveness of using drug susceptibility testing and second-line drugs in a lower-middle-income setting with high levels of MDR TB.

**Methods and Findings:**

We developed a dynamic state-transition model of TB. In a base case analysis, the model was calibrated to approximate the TB epidemic in Peru, a setting with a smear-positive TB incidence of 120 per 100,000 and 4.5% MDR TB among prevalent cases. Secondary analyses considered other settings. The following strategies were evaluated: first-line drugs administered under directly observed therapy (DOTS), locally standardized second-line drugs for previously treated cases (STR1), locally standardized second-line drugs for previously treated cases with test-confirmed MDR TB (STR2), comprehensive drug susceptibility testing and individualized treatment for previously treated cases (ITR1), and comprehensive drug susceptibility testing and individualized treatment for all cases (ITR2). Outcomes were costs per TB death averted and costs per quality-adjusted life year (QALY) gained. We found that strategies incorporating the use of second-line drug regimens following first-line treatment failure were highly cost-effective compared to strategies using first-line drugs only. In our base case, standardized second-line treatment for confirmed MDR TB cases (STR2) had an incremental cost-effectiveness ratio of $720 per QALY ($8,700 per averted death) compared to DOTS. Individualized second-line drug treatment for MDR TB following first-line failure (ITR1) provided more benefit at an incremental cost of $990 per QALY ($12,000 per averted death) compared to STR2. A more aggressive version of the individualized treatment strategy (ITR2), in which both new and previously treated cases are tested for MDR TB, had an incremental cost-effectiveness ratio of $11,000 per QALY ($160,000 per averted death) compared to ITR1. The STR2 and ITR1 strategies remained cost-effective under a wide range of alternative assumptions about treatment costs, effectiveness, MDR TB prevalence, and transmission.

**Conclusions:**

Treatment of MDR TB using second-line drugs is highly cost-effective in Peru. In other settings, the attractiveness of strategies using second-line drugs will depend on TB incidence, MDR burden, and the available budget, but simulation results suggest that individualized regimens would be cost-effective in a wide range of situations.

## Editors' Summary

**Background.:**

Tuberculosis (TB) remains one of the most entrenched diseases on the planet—an estimated one in three people worldwide are infected with *Mycobacterium tuberculosis,* which causes the disease. Although effective drugs exist, a major reason for the failure to stem the spread of TB lies in the rise of drug-resistant strains of the bacterium. Some strains are resistant to several drugs; patients with this sort of infection are said to have multidrug-resistant (MDR) TB. The development of drug-resistant strains is fostered when health-care workers do not follow treatment guidelines or fail to ensure that patients take the whole treatment course. The World Health Organization recommends an approach to TB control called “DOTS,” which has been adopted by many countries. (See the link below for an explanation of what DOTS involves.) The antibiotics that are used in DOTS are described as “first-line” treatment drugs. They are highly effective against non-resistant TB but much less so against MDR TB. There are other, more expensive, “second-line” antibiotics that perform better against MDR TB.

**Why Was This Study Done?:**

Despite the worrying rise in MDR TB cases, the much higher cost of using second-line drugs is prompting some policy-makers to question the merits of introducing them in poor countries with limited resources. However, with MDR TB accounting for nearly a third of TB cases in some countries, first-line therapies seem unlikely to be sufficient in the long term. Second-line strategies, or “DOTS-Plus” strategies, are either standardized for a particular region or are chosen for individual patients on the basis of drug susceptibility tests. The researchers wanted to investigate whether standardized or individualized second-line regimens could save lives and be cost-effective in poor countries.

**What Did the Researchers Do and Find?:**

The researchers used a method called modeling. They took information already available about TB in Peru, where for every 100,000 people there are 120 new TB infections every year, and 4.5% of existing cases are MDR TB. The researchers then calculated what might happen over the next 30 years, comparing the likely effects of five alternative strategies. In four, new cases were given first-line drugs for 6 months. Those who were not cured were then treated in different ways. In DOTS, they were retreated with a second course of the same drugs; in STR1 they were given an 18-month standardized course of second-line and first-line drugs; in STR2, only confirmed MDR TB patients were given an 18-month standardized course of second-line and first-line drugs; and in ITR1, confirmed MDR TB patients were given a personalized regimen of second-line drugs. The fifth strategy, ITR2, tested all patients for drug susceptibility at the outset of treatment, and those with MDR TB were given an individualized course; those not cured were tested again and given another individualized course.

Compared with DOTS, both the STR1 and STR2 strategies averted 4.8 deaths per 100,000 population, at a cost of $8,700 per averted death—with STR2 being a better value for money since it treated only confirmed MDR TB cases with the more expensive, second-line drugs. Of the individualized treatments, ITR1 averted an extra 0.9 deaths at a cost of $12,000 per averted death; ITR2 averted a further 1.2 deaths but at a much higher $160,000 per saved life.

**What Do These Findings Mean?:**

Despite the slightly higher cost of ITR1, the extra number of lives it would save compared with STR2 makes it a good approach for treatment in Peru. However, cost-effectiveness varies with other factors. If the difference in cost between the two strategies became higher than $9,500 per patient, STR would be preferable. And, if MDR TB were present in 10% of all TB cases, ITR2—with comprehensive drug susceptibility testing for all TB patients—would be best.

The findings are of interest not just in Peru but in other developing countries where MDR TB is a growing problem. The researchers maintain that, in areas where DOTS has not yet been fully implemented, it would be more efficient to expand DOTS than to introduce DOTS-Plus. But they add that it would be beneficial to expand DOTS as well as implement DOTS-Plus. Individualized treatment after drug susceptibility testing is likely to be cost-effective even in the poorest of countries, which should give impetus to governments and organizations in those countries where MDR TB is a growing concern to modify their approach to treatment.

**Additional Information.:**

Please access these Web sites via the online version of this summary at http://dx.doi.org/10.1371/journal.pmed.0030241.

• Basic information about tuberculosis can be found on the Web site of the US National Institute of Allergy and Infectious Diseases

• The Web site of the World Health Organization's Stop TB department outlines both the DOTS and DOTS-Plus strategies

• TB Alert, a UK-based charity that promotes TB awareness worldwide, has information on TB in several European, African, and Asian languages

## Abstract 3

Resch and colleagues found that treatment of MDR TB using second-line drugs is highly cost-effective in Peru.

## Introduction

Mycobacterium tuberculosis infects nearly one-third of the world's population, and 8 to 10 million infected persons progress to active tuberculosis (TB) each year [ 1]. Despite the existence of effective drug treatment, TB causes approximately 2 million deaths annually [ 1]. Efforts to treat patients with active disease and to control the spread of TB are complicated by resource constraints, co-infection with HIV, and the emergence of drug-resistant TB strains.

Multidrug-resistant TB (MDR TB), defined by resistance to the two most potent first-line anti-TB agents (isoniazid and rifampicin), arises initially as a result of poorly implemented treatment. Subsequent transmission of MDR TB strains gives rise to cases of “primary” resistance. Among 77 settings surveyed by the World Health Organization (WHO), the estimated fraction of prevalent TB cases that are MDR ranged from 0% to 27% (median 1.7%) [ 2].

The optimal strategy for detecting and treating MDR TB in resource-poor countries is unclear. Directly observed therapy using a standard short course of first-line antibiotics (DOTS), as developed and promoted by WHO, is widely endorsed by national TB programs. Several studies report high cure rates using DOTS for drug-sensitive TB [ 1, 3– 6]. However, DOTS has been shown to be much less effective against MDR TB, with cure rates in six countries ranging from 6% to 59% [ 6].

Treatment strategies that include the use of second-line drugs in the directly observed treatment of MDR TB (DOTS-Plus) can achieve cure rates nearly as high as those for drug-sensitive TB treated with first-line drugs [ 7, 8]. Strategies for using second-line drugs fall into two broad categories: those using standardized regimens formulated for particular geographic areas based on drug resistance profiles of a sample of cases, and those using individualized regimens selected on the basis of individual drug susceptibility testing (DST).

Although second-line therapy yields higher cure rates for MDR TB, it is more expensive than first-line therapy and requires longer treatment durations. Policy-makers have questioned the wisdom of allocating resources to second-line therapy, particularly where DOTS programs are not fully implemented [ 9].

Our objective was to assess the health benefits and cost-effectiveness of identifying and treating patients with MDR TB in lower-middle-income settings using the example of Peru, where an estimated 4.5% of all TB cases are MDR TB [ 10, 11]. Our analysis differs from previously published cost-effectiveness analyses [ 12, 13] in that it uses more recent data on the efficacy of DOTS-Plus and uses a dynamic model to simulate the reduction of TB transmission in the community as a benefit of treatment.

## Methods

We used a state-transition model to evaluate the cost-effectiveness of five alternative treatment strategies for TB. Strategies were evaluated in terms of incremental costs per averted TB death and incremental costs per quality-adjusted life year (QALY) saved. The model is similar to a Markov cohort model, but allows the incidence of drug-susceptible TB and MDR TB infection to depend on the current prevalence of infectious cases in the population. Model parameters were calibrated to represent the current TB epidemic in Peru. Co-infection with HIV was not explicitly considered because only 2% of TB cases in Peru have HIV co-infection [ 10]. The time horizon for the analysis was 30 y, and the perspective was that of a public health-care system.

### Treatment Strategies

The following strategies were considered.

#### DOTS.

New cases are treated with a 6-mo course of first-line drugs, and previously treated patients who are not cured are retreated with a second course of first-line drugs.

#### STR1.

New cases are treated with first-line drugs, and previously treated patients who are not cured receive an 18-mo standardized regimen of three second-line drugs and two first-line drugs.

#### STR2.

New cases are treated with first-line drugs, and previously treated patients are tested for MDR TB. Confirmed cases receive an 18-mo standardized regimen of three second-line drugs and two first-line drugs.

#### ITR1.

New cases are treated with first-line drugs, and previously treated patients who are not cured receive comprehensive DST; those with confirmed MDR TB receive an individualized regimen of second-line drugs.

#### ITR2.

New and previously treated patients receive DST, and those with MDR TB receive an individualized regimen; those not cured are given a repeat DST and another individualized treatment regimen.

In all strategies, patients who are not cured by two courses of treatment continue to receive treatment, which is assumed to reduce their mortality risk but confer no added probability of cure.

### Model Structure and Simulation

**Figure 1:**
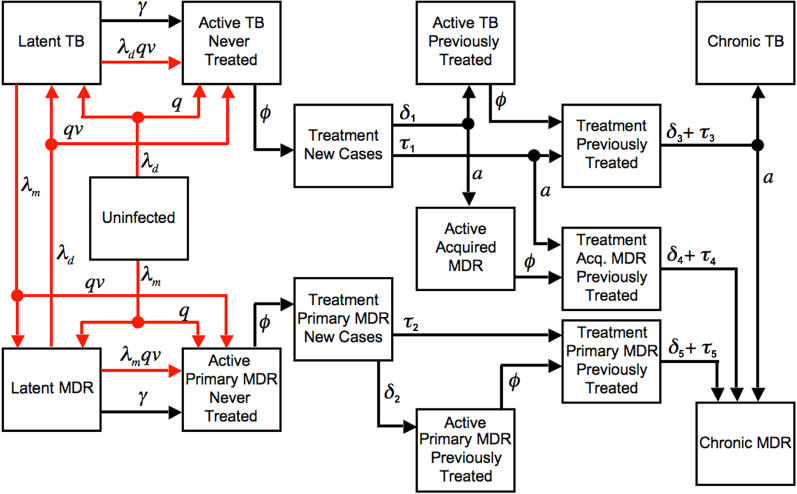
Structure of the TB Treatment Model Boxes represent health states, arrows represent population flow between health states, red arrows represent infection and re-infection. λ _d_ is the force of non-MDR infection, λ _m_ is the force of MDR TB infection, *q* is the proportion of new infections that break down rapidly, *v* is the immunity factor, γ is the rate of delayed progression from latent to active disease, ϕ _i_ is the case detection rate, δ _i_ is the treatment dropout rate, τ is the treatment failure rate, and *a* is the fraction of uncured patients acquiring MDR. Death can occur from any state (not shown). Cure can occur from any diseased state. Cured patients transition to the latent infection health state (not shown).

The model ( Figure 1) begins with a population of 100,000 people, distributed across health states to distinguish uninfected from infected persons, latent infection from active disease, non-MDR from MDR infection, and various treatment histories (see Text S1 and Tables S1– S3 for technical details). In each monthly cycle persons may transition from one state to another, reflecting the processes of infection, progression, treatment initiation and completion, and mortality. Active disease is limited to smear-positive pulmonary cases, since they are the primary target of DOTS-based TB control strategies.

### Natural History

Transmission of TB infection from persons with active disease to those who are uninfected or latently infected increases proportionally with the number of infectious persons in the population at any specific time. In the base case we assumed that MDR TB is as transmissible as drug-susceptible TB [ 14– 16]. In the model, a proportion of new infections progress directly to the active disease state, and the remainder enter a latent state and are subject to subsequent activation at a constant rate [ 17, 18]. Without treatment, patients may cure spontaneously, remain actively infected, or die. Natural history assumptions ( Table 1) were derived from epidemiologic studies [ 14, 17– 24] and by calibrating the model to produce estimates consistent with epidemiologic data from Peru (see Text S1 and Table S4). Since more than one combination of model parameters could generate an epidemic consistent with available data, several sets of parameter combinations were considered in sensitivity analysis.

**Table 1:**
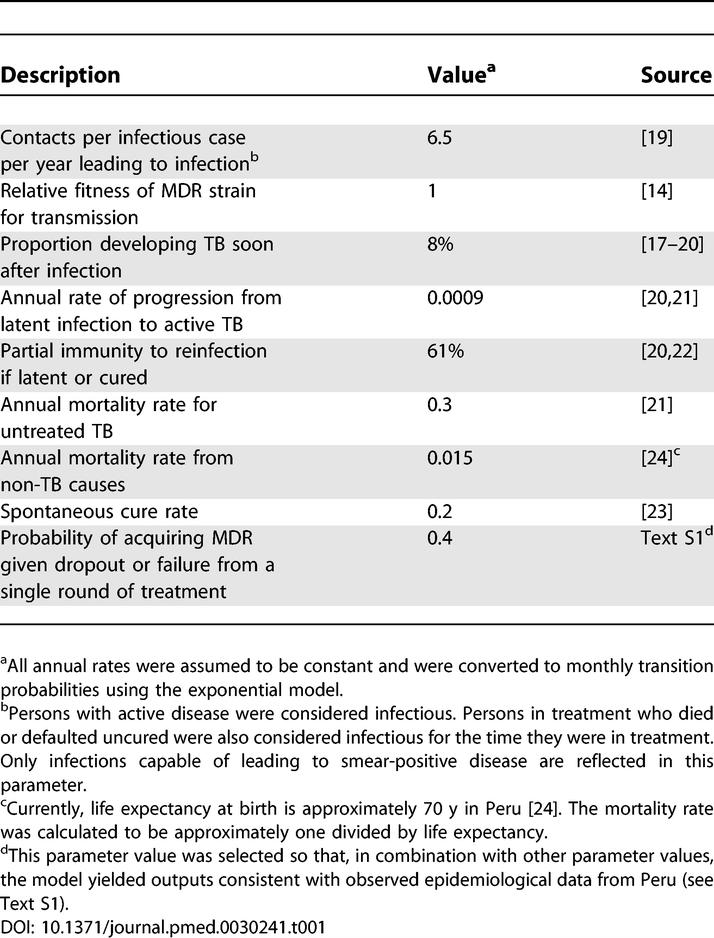
Natural History Assumptions

### Diagnosis and Treatment

New TB cases are detected when patients present with pulmonary symptoms and are diagnosed by sputum smear microscopy. Consistent with the high rates of case detection achieved in Peru, we assumed that a new TB case had an 80% annual probability of being detected and entering treatment [ 3]. However, WHO estimates that, on average, in settings with fully implemented DOTS programs, only about half of incident TB cases are detected annually using passive case detection [ 25]. Therefore, in sensitivity analysis we examined the impact of the case-finding rate on outcomes.

Probabilities of cure, default (dropout), and death in each treatment strategy listed in Table 2 are based on published studies of treatment cohorts [ 3– 7, 13, 26– 33]. First-line DOTS regimens cure approximately 90% of new drug-susceptible cases, and reduce mortality to less than 5% [ 3– 6]. Default rates vary, but usually fall below 10% in well-administered programs [ 3– 6]. In contrast to the high cure rates among new cases, between 57% and 88% of non-MDR cases are cured by retreatment regimens [ 6, 31, 32]; we assumed a cure probability of 68% in our base case. First-line drugs are even less effective against MDR TB [ 6, 31]; for our base case, we assumed a cure probability of 58% for new MDR TB cases treated with a first-line regimen and 35% for retreatment.

**Table 2:**
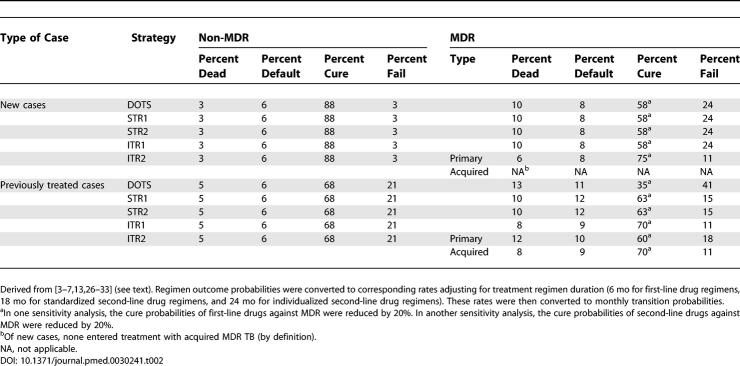
TB Treatment Outcome Parameters

Studies of standardized second-line drug regimens have reported cure probabilities ranging from 44% to 72% [ 13, 26– 28]. For our base case, we assumed a 63% probability of cure, based on an average of these results. Studies of individualized treatment have reported success rates of 75% in the United States [ 30], 77% in Turkey [ 29], and 73%–79% in Peru [ 7, 33]. We assumed 75%, 70%, and 60% probabilities of cure for previously untreated patients, patients for whom a first-line regimen had failed, and patients for whom a second-line regimen had failed, respectively.

Poor adherence and other factors introduce a risk of acquired MDR TB for patients in treatment; thus, the model allows for 40% of treatment failures and uncured defaulters to acquire MDR TB. In the model, cured cases remain latently infected, and can reactivate or be reinfected.

### Costs

Regimen costs include the cost of drugs, laboratory tests, and personnel ( Table 3) [ 13, 34]. Patient time and transportation costs are excluded. Base case drug costs for second-line regimens and DST are based on unpublished information obtained from Partners In Health, a Boston-based nongovernmental organization delivering TB treatment in Peru (see Text S1 and Table S5 for details). Non-drug costs for second-line regimens are based on a previously published cost-effectiveness analysis [ 13]. Published estimates of first-line regimen costs are available for several settings. [ 4, 13, 34– 36]. For consistency, we used the estimates of Suarez et al. [ 13] in our base case. We assumed that patients who default or die during treatment incur half the costs of those completing treatment [ 13]. All costs are reported in 2004 US dollars.

**Table 3:**
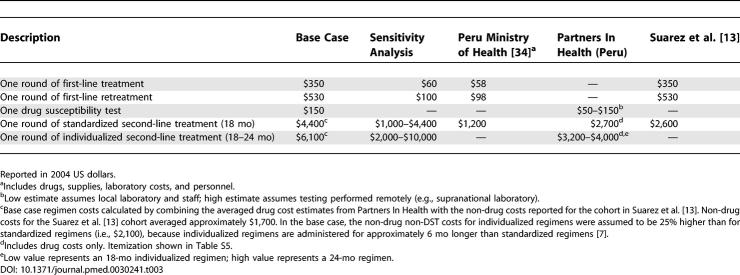
Treatment Costs

### Health State Utilities

For calculation of QALYs, we use a utility weight of 0.58 for active TB, based on a WHO study [ 37]. Other health states are assigned a weight of 0.85, based on age-specific health state values of Peruvian adults weighted by the current age distribution [ 38, 39].

### Cost-Effectiveness Analysis

Future costs and health outcomes are discounted to present value at an annual rate of 3% [ 40, 41]. Strategies are ranked in order of increasing cost, and any strategy that is more costly and less beneficial than another strategy is considered dominated. The incremental cost-effectiveness ratio for each remaining strategy is calculated by dividing the additional cost compared to the next most costly strategy by the additional benefit (QALYs gained or TB deaths averted). The Commission on Macroeconomics and Health suggests that interventions costing less than three times the gross domestic product (GDP) per disability-adjusted life year (DALY) averted may be considered good value [ 42]. (One DALY averted is analogous to one QALY gained.) This benchmark is endorsed by WHO [ 43]. In our evaluation, we considered an incremental cost per QALY that was less than the per capita GDP to be highly cost-effective, and we considered three times the per capita GDP to be a threshold beyond which an intervention would be considered too expensive. The per capita GDP in Peru is $2,360 [ 24].

### Sensitivity Analyses

Because there is variation in and uncertainty about the effectiveness and cost of MDR TB treatment, we performed several sensitivity analyses to determine the stability of our base case findings. Parameter values for these analyses are reported in Table S4. The MDR TB cure probabilities associated with each regimen were varied, with probabilities of other treatment outcomes adjusted proportionately. We also explored the sensitivity of our results to uncertainty in regimen cost. To characterize the tradeoffs between STR2 and ITR1, we simultaneously varied the differential MDR TB cure probability and the differential cost between standardized and individualized regimens.

The underlying biology and epidemiological characteristics of MDR TB are also uncertain. In sensitivity analyses we considered alternative assumptions about TB transmission and progression, including an assumption that MDR TB strains are only half as transmissible as non-MDR strains. We also considered alternative settings that differ from Peru in terms of the magnitude of the overall TB epidemic in the population, the fraction of prevalent TB cases that are MDR, and the efficacy of case-finding.

We performed another sensitivity analysis that excluded reductions in transmission associated with successful treatment of TB or MDR TB in order to distinguish the direct benefit of treatment to patients from the indirect benefit to the community through transmission reduction. In this analysis, both the incidence of infection and the fraction of primary MDR TB among new cases were held constant during the intervention period.

Lastly, we considered a multivariable “worst-case scenario” in which several parameter values were biased against second-line treatment strategies: the cost of first-line regimens were reduced to $60 for new cases and $100 for retreatment, the effectiveness of second-line regimens against MDR TB was 20% lower, the relative transmissibility (fitness) of MDR TB was 50%, and the time horizon was only 10 y.

## Results

### Base Case

The discounted costs, discounted benefits, and incremental cost-effectiveness ratios for the alternative strategies are shown in Table 4. Incidence trends are reported in Figure S1. Over the 30-y time horizon, both standardized treatment strategies (STR1 and STR2) averted 4.8 deaths and gained 59 QALYs per 100,000 persons compared to the DOTS strategy. STR2, which utilized a test for MDR TB to screen out false positives, provided this incremental benefit at lower cost than STR1, and had an incremental cost-effectiveness of $720 per QALY ($8,700 per averted death) compared to the DOTS strategy. The strategy incorporating comprehensive DST for previously treated cases and individualized second-line treatment for MDR TB (ITR1) saved an additonal 12 QALYs (0.9 deaths) per 100,000 persons compared to STR2, at an incremental cost of $990 per QALY gained ($12,000 per averted death). A more aggressive strategy that extended DST to new TB cases as well (ITR2) saved an additional 16 QALYs (1.2 deaths) compared to ITR1, for an incremental cost-effectiveness of $11,000 per QALY ($160,000 per averted death). Figure S2 shows the efficiency frontier for the base case strategies.

**Table 4:**
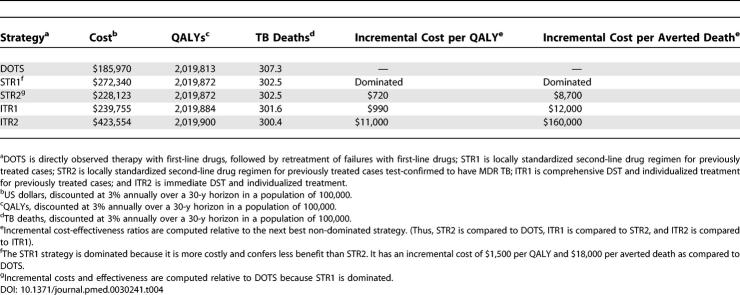
Incremental Cost-Effectiveness of Second-Line Treatment Strategies for MDR TB: Base Case Results for Peru

In Peru, with a population of 27 million, DOTS-Plus with standardized regimens (STR2) would avert 2,010 (undiscounted) deaths and individualized regimens (ITR1) would avert 2,400 deaths as compared to DOTS alone over a 30-y period at an (undiscounted) cost of $17 million and $21 million dollars, respectively.

### Sensitivity Analyses

Table 5 reports the results of several sensitivity analyses. Additional sensitivity analysis results are presented in Table S6. When outcomes are measured over a 30-y time horizon, the incremental cost-effectiveness of both STR2 and ITR1 remains under $1,900 in all one-way sensitivity analyses. In the worst-case scenario, STR2 is dominated and the cost-effectiveness of ITR1 is $6,400 per QALY compared to DOTS.

**Table 5:**
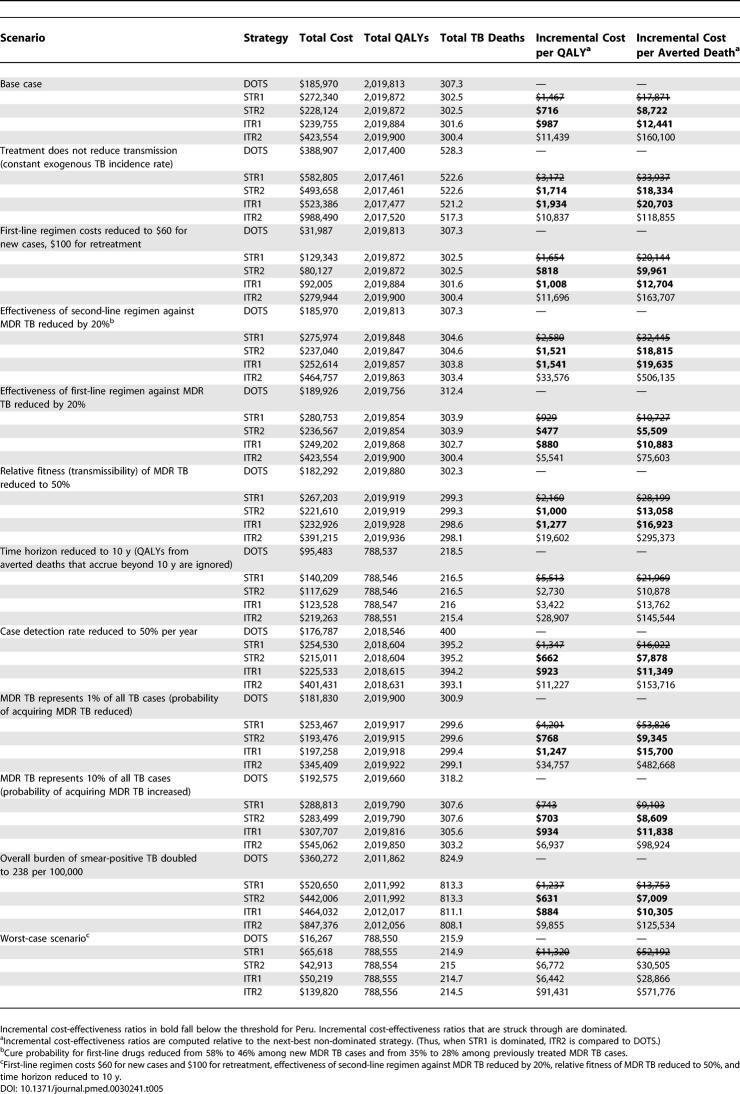
Sensitivity Analyses

Among the non-dominated strategies (DOTS, STR2, ITR1, and ITR2), ITR1 would be selected for Peru using three times the per capita GDP as a threshold for cost-effectiveness. Although the relative performance of ITR1 compared to STR2 was sensitive to assumptions about the MDR TB cure probability and cost of each regimen, the ITR1 remained the optimal choice within plausible ranges for these parameters. If cure probabilities are 7% lower in standardized compared to individualized regimens (as in our base case), the difference in cost between standardized and individualized regimens would have to exceed $9,500 in order for STR2 to be the preferred strategy. If cure probabilities for standardized regimens are just 2% lower than those for individualized regimens, STR2 would be preferred to ITR1 if the difference in regimen costs exceeded $3,200.

The incremental cost-effectiveness of the ITR2 strategy compared to ITR1 improves significantly when first-line drugs are less effective against MDR TB and when the fraction of MDR TB cases among all cases is increased to 10%.

## Discussion

Our cost-effectiveness analysis of treatment strategies provides evidence that DOTS-Plus strategies are likely to be cost-effective in lower-middle-income settings with at least 1% MDR TB. Over a wide range of assumptions, a strategy of testing previously treated patients for MDR TB and then treating them with second-line regimens was found to be cost-effective compared to first-line DOTS alone. In our base case for Peru, we estimate that STR2 would avert 4.8 deaths per 100,000 persons over 30 y, at an incremental cost of $720 per QALY compared to DOTS. A policy of comprehensive DST and individualized treatment for first-line failures (ITR1), the best performing strategy under the cost-effectiveness threshold of the per capita GDP, averted 0.9 additional deaths per 100,000 at an incremental cost of $990 per QALY compared to STR2. A clinical strategy that begins with comprehensive DST for all cases upon diagnosis of TB may be cost-effective in settings with high levels of MDR or where first-line drugs perform especially poorly against MDR TB. The base case incremental cost per QALY gained with this strategy (ITR2 compared to ITR1) was $11,000, but when the prevalence of MDR TB among prevalent TB cases was 10%, the cost per QALY gained for ITR2 dropped to $6,400. Poorer performance of first-line drugs against MDR TB increases the potential gains from immediate diagnosis and treatment of MDR TB. When the effectiveness of a first-line regimen was dropped 20% from the base case assumptions, the ITR2 strategy had a cost per QALY of $5,500.

In our analysis, DOTS-Plus based on standardized second-line regimens administered to previously treated patients presumed to have MDR TB (STR1) was consistently dominated because it provided the same benefit as STR2 at a higher overall cost. This result is largely due to the costs of treating non-MDR cases unnecessarily with second-line therapy. In the base case calibration of our model, on average 54% of cases entering a second round of treatment had MDR TB. This figure is consistent with the outcome of a large retrospective study of laboratory results in Peru that found that 57% of previously treated patients reentering treatment had MDR TB [ 44].

DOTS-Plus programs that use standardized regimens typically do not use DST to determine each patient's resistance profile [ 26, 27, 45], but in a South African DOTS-Plus program, patients suspected of having MDR TB are tested for first-line drug resistance before standardized second-line regimens are administered [ 46]. Although the STR2 strategy, which mimics the South African program, is an efficient strategy, we found that ITR1 conferred additional benefit at an incremental cost of $990 per QALY, which is still well below the per capita GDP of Peru. In Peru, the difference in MDR TB cure probability between individualized and standardized regimens must be less than 2%, and standardized regimens must be $3,200 less expensive, in order for the incremental cost-effectiveness ratio of ITR1 to be considered beyond the threshold of three times the GDP ($7,080).

The finding that individualized second-line treatment of previously treated cases (ITR1) is highly cost-effective was stable over a wide range of assumptions. The incremental cost per QALY gained for ITR1 remained under $1,900 in all one-way sensitivity analyses. When considering a situation in which several one-way sensitivity analyses were combined into a “worst-case scenario” over a 10-y time horizon with several parameter values simultaneously biased against second-line treatment strategies, STR2 is dominated, but ITR1 still appears quite favorable at $6,400 per QALY as compared to DOTS alone. The incremental cost-effectiveness ratio of the more aggressive individualized treatment strategy (ITR2) compared to ITR1 exceeded the threshold of three times the per capita GDP in the base case, but appeared more favorable when the fraction of MDR TB among prevalent TB cases was higher, and also under the plausible assumption that the cure probability for first-line therapy against MDR TB is 20% lower than assumed in the base case.

Interrupting transmission is a critical component of the overall benefit of treatment. When these community-level benefits were excluded, costs per QALY or per averted death by STR2 and ITR1 approximately doubled. Nonetheless, individualized treatment with second-line drugs remained cost-effective by international standards even without accounting for transmission benefits.

A key uncertainty in our model is the relative fitness of MDR TB strains for transmission. Recent models [ 15, 16] indicate that over very long time horizons, heterogeneous fitness among MDR strains will lead to the eventual dominance of MDR TB as long as some fraction of MDR strains are as fit as drug-sensitive strains. We assumed that MDR TB was equally transmissible as drug-susceptible TB in our base case, but considered the possibility that it is up to 50% less transmissible. When MDR TB fitness was reduced, DOTS-Plus strategies became somewhat less cost-effective, but remained attractive.

We assumed that second-line therapies were no more effective than DOTS in TB patients without MDR TB. This assumption may underestimate the benefits of DOTS-Plus strategies if second-line drugs confer additional benefits in patients with non-MDR drug resistance.

The analysis reflects the perspective of a public health-care system and includes the costs of medical care only. An analysis from the societal perspective would need to incorporate costs for patient time, transportation, and unpaid caregiver time, but such data were not available and would be unlikely to alter the conclusions.

An analysis by Sterling et al. [ 12] found that a DOTS-Plus strategy using DST and second-line drugs over a 10-y time period had an incremental cost-effectiveness ratio of $68,860 per averted death compared to a DOTS strategy using first-line drugs only, suggesting that it would be relatively expensive for low- to middle-income countries. However, the study used estimates of the effectiveness of second-line therapy that are lower than current data suggest [ 7, 13, 26– 30, 33] and assumed that TB incidence and the fraction of MDR TB among new cases would not decline in response to treatment.

Suarez et al. [ 13] found that the use of second-line drugs in patients failing other therapies had an incremental cost-effectiveness ratio of about $200–$700 per DALY averted (year 2000 US dollars). Our dynamic transmission modeling results corroborate the finding of Suarez et al. [ 13] that second-line therapy for MDR TB patients is highly cost-effective in Peru.

Like Sterling et al. [ 12] and Suarez et al. [ 13], our study suggests that DOTS-Plus is both more effective and more costly than DOTS. Therefore, if DOTS has not been fully implemented and can be expanded within the available infrastructure, it would be more efficient to expand DOTS coverage than to initiate DOTS-Plus. Similarly, an implementation of DOTS-Plus that uses non-surplus resources from an existing DOTS program would also be inefficient. Nevertheless, our results also show that fully implementing DOTS and initiating DOTS-Plus would be a reasonable use of resources in many settings.

We quantified the marginal gains of relatively more aggressive strategies to control MDR TB and found that the relatively high cost of second-line therapy (as compared to first-line therapy) should not be perceived as a barrier to implementation of DOTS-Plus programs. We did not account for start-up costs (e.g., laboratory capacity) required for DST. However, in lower-middle-income countries such as Peru, with about 2,900 MDR TB cases each year [ 47], even if a lump sum of $5.5 million for start-up costs were added in the first time period to the costs of ITR1, that strategy would still have a cost per QALY less than the per capita GDP.

We found that standardized regimens could be cost-effective when a test for MDR TB is used before enrolling previously treated patients into second-line therapy, suggesting the possible utility of an inexpensive rapid test for MDR such as the Greiss method [ 48]. We also found that comprehensive DST for previously treated patients followed by individualized treatment for MDR TB cases will likely be cost-effective in a variety of settings, even in countries with severely constrained resources. Furthermore, immediate DST for all detected TB cases and individualized second-line treatment for MDR TB may be cost-effective in middle-income countries with high levels of MDR TB.

The feasibility of delivering effective individualized TB care has been demonstrated in Peru and other countries. Our study finds that the strategy of testing previously treated cases with DST and treating MDR TB with individualized regimens would be cost-effective in Peru under a wide range of alternative assumptions about treatment costs, effectiveness, MDR TB prevalence, and transmission, including a range of assumptions regarding the relative performance of a similar strategy based on standardized regimens. Our study contributes to a growing body of evidence that indicates that national TB programs and nongovernmental organizations should move quickly to implement DOTS-Plus in settings where multiple drug resistance is prevalent.

## Supporting Information

Figure S1 Incidence Trends Annual non-MDR and MDR TB incidence per 100,000 persons under the four non-dominated TB control strategies in the base case. MDR TB is initially generated during the substandard treatment era that precedes the 30-y intervention era. MDR TB incidence initially declines under all four control strategies, but when only first-line treatment is available, the decline reverses after a few years.
(10.1 MB TIF)

Figure S2 Efficiency Frontier for MDR TB Control Strategies In the base case, in order of increasing effectiveness and cost, DOTS, STR2, ITR1, and ITR2 lie along the efficiency frontier. STR1 is dominated because it costs more than STR2 and provides no more benefit.
(460 KB JPG)

Table S1 Treatment Outcome Parameters for Substandard Treatment Era (32 KB DOC)

Table S2 Description of Model States (63 KB DOC)

Table S3 Description of Model Parameters (139 KB DOC)

Table S4 Calibration to Peru and Parameters Used in Sensitivity Analysis (57 KB DOC)

Table S5 Itemized Cost Calculations for Regimens Using Second-Line Drugs (91 KB DOC)

Table S6 Sensitivity Analyses (Extended Version of Table 5)
(191 KB DOC)

Text S1 Appendix (1.2 MB DOC)

## Abbreviations

DALY: disability-adjusted life year
DST: drug susceptibility testing
GDP: gross domestic product
MDR: multidrug-resistant
QALY: quality-adjusted life year
TB: tuberculosis
WHO: World Health Organization
