# Supplementary material for: Cost-Effectiveness of Treating Multidrug-Resistant Tuberculosis
Source: PLoS Med. 2006 Jul 4;3(7):e241. doi: 10.1371/journal.pmed.0030241 (PMC1483913; doi:10.1371/journal.pmed.0030241)
Supplement: Supplementary file 1 [file pmed.0030241.sd001.doc]

### Supplementary Material

### Analytic Overview

We developed a dynamic state-transition model to estimate the incremental cost per averted TB death and the incremental cost per quality-adjusted life year (QALY) gained under different strategies for treating MDR-TB. The model is dynamic in that it permits the incidence of new drug-susceptible and MDR-TB cases to depend on transmission from prevalent cases. For the base case, the model was calibrated to represent the current TB epidemic in Peru. Coinfection with HIV was not explicitly considered because only 2% of TB cases in Peru are coinfected [1]. The time horizon for the analysis is 30 years, and the perspective is that of a public health care system.

**Treatment Strategies**

We evaluated five treatment strategies. The first (DOTS) is the basic DOTS treatment protocol widely implemented in resource-poor settings. New patients receive a 6-month regimen of first-line drugs which begins with 2 months of rifampin, isoniazid, ethambutol, and pyrazinamide, followed by 4 months with rifampicin and isoniazid. Patients in whom this regimen fails are retreated with the same 4 first-line drugs plus streptomycin. Under this strategy no drug susceptibility testing is performed and no second-line drugs are prescribed for MDR-TB cases. The second (STR1) is a DOTS-Plus strategy in which all new cases are treated with first-line drugs, and patients who have been previously treated but not cured are presumed to have MDR-TB and receive an 18-month standardized regimen of three to five second-line drugs and two first-line drugs. The third strategy (STR2) is similar, but uses a test to confirm resistance to rifampin and isoniazid before starting a regimen of second-line drugs in previously treated patients. The studies used to inform treatment outcome parameters represent a variety of standardized treatment protocols. Our estimates are intended to represent the average performance of standardized treatment regimens. The third (ITR1) is also a DOTS-Plus strategy, but patients who have been previously treated but not cured receive comprehensive drug susceptibility testing (DST). Patients with laboratory-confirmed MDR-TB are then given an 18-24 month individualized regimen of second-line drugs based on their specific resistance profile. Because studies of individualized regimens frequently report median duration near 24 months (e.g. [2], [3]), we assumed a 24 month duration for these regimens. Under the fourth, and most aggressive, strategy (ITR2), all patients get DST prior to any treatment. Patients with laboratory-confirmed MDR-TB are treated with an individualized regimen, and those not cured by the first course of individualized treatment are given a repeat DST and another course of individualized treatment regimen. Particular settings may incorporate different second-line drugs into their DOTS-Plus regimens. Our estimates of regimen performance represent an average of published results DOTS-Plus program using individualized regimens. Under both individualized strategies, patients not cured by the first course of treatment but who do not have MDR-TB are retreated with first-line drugs. Under all treatment strategies, new TB cases are detected when patients present with pulmonary symptoms and are diagnosed by sputum smear microscopy. Also, under all four strategies, patients who are not cured by two courses of treatment are considered chronic cases and continue to receive the same regimen type that they most recently received, which is assumed to reduce their mortality risk but confers no added probability of cure.

**Costs**

###	The per-patient cost of community-based first-line DOTS has been reported for several settings including Peru [4,5], Russia[6], and sub-Saharan Africa[7,8]. Similarly, the costs for STR were reported for Peru in two separate studies[4,5]. However, the total cost of treating MDR-TB in Peru patients with individualized regimens has not been published. Costing of ITR is further challenged by variation in drugs and dosages used in second-line regimens as well as changes in price of second-line drugs since the WHO Green Light Committee negotiated price concessions. Therefore drug costs for individual and standardized regimens were based on estimates calculated by the Partners In Health organization for regimens they use to treat patients in Peru. The itemized costs estimates are shown in Table S1. The aggregate estimates are calculated according to the proportion of patients using each of a battery of second line agents. The per-unit drug costs are based on WHO Green Light Committee negotiated prices.

The cost of comprehensive DST was obtained through Partners in Health and the Massachusetts State Laboratory. It is based on lab samples collected in Peru being sent to a US laboratory for processing. A limited DST used to identify MDR-TB (resistance to rifampin and isoniazid) was assumed to be half the cost of a comprehensive DST. We did not explore testing protocols in detail because our results were not sensitive to testing costs within plausible ranges.

### Model structure

Figure 1 (main text) displays the general structure of the model. The model is composed of four main health states: uninfected, latent *M. tuberculosis* infection, untreated active TB, and treated active TB. The active TB states are further subdivided to track the drug susceptibility of the infecting strain and treatment history. The latent infection state is subdivided to track drug susceptibility, but not treatment history. Drug susceptibility is categorized as either MDR-TB, defined as resistance to at least rifampicin and isoniazid, or non-MDR-TB (which includes strains resistant to no drugs, one drug, or more than one drug but not including both rifampicin and isoniazid). We also distinguished between primary and acquired MDR-TB so that, under the ITR2 strategy, we could assign a higher treatment success probability of newly acquired MDR-TB that was being treated with second-line drugs in the second course of treatment than for primary cases of MDR-TB who were being treated with second-line drugs for a second time. Treatment history is defined as either “none” or “previously treated.” The model is limited to smear-positive cases, since they are the primary target of DOTS-based TB control strategies, and because smear-negative disease in the HIV-uninfected population has lower mortality [9,10] and was recently estimated to be only about 20% as infectious [11].

The model begins with a population of 100,000 people, distributed across health states to reflect current prevalence and treatment history. The passage of time is simulated in one-month cycles. In each cycle, proportions of the population move among health states according to transition probabilities, new uninfected persons enter the model, and persons may die of TB or other causes and exit the model. The equations governing these transitions are available upon request.

**Model Transitions**

*Incidence.* In the base case, TB incidence is based on transmission from persons with active disease to uninfected or latently infected persons, with the risk of infection proportional to the number of infectious persons. All individuals with untreated active disease and a fraction of those in treatment are considered infectious.

*Progression.* Of new infections, a proportion progress directly to the active disease state; the remainder enter a latent state and are subject to subsequent activation at a constant rate. This dichotomy of “fast” versus “slow” progression approximates the observed clinical progression of TB, in which the risk of developing active disease diminishes exponentially over time with nearly 10% of new infections becoming active within the first 2 years. [12, 13]. We assume that those with untreated active disease may cure spontaneously at a constant rate. We further assume that having latent infection offers partial protection against progression to active disease upon reinfection [14], and that the strain of a new infection (non-MDR or MDR) supersedes the strain of a pre-existing latent infection.

*Case finding.* In Peru, about 80% of incident TB cases are detected per year. [15] We assume that a fraction of active cases are detected and enter treatment each month, at a rate consistent with this estimate. However, WHO estimates that, on average, in settings with fully implemented DOTS programs, only about half of TB patients are detected annually using passive case detection [16]. Therefore, in sensitivity analysis we considered this case finding rate as well.

*Treatment.* Being in treatment is assumed to protect fully against reinfection due to ongoing chemotherapy. Persons in treatment can exit that state by cure, default (dropout), failure, or death. We assume that one-third of defaulters would have been on treatment long enough to achieve the same probability of being cured as those completing treatment. A portion of treatment failures and uncured defaulters acquire MDR-TB. Cured cases remain latently infected, and can later reactivate or be reinfected, at which point they are considered new cases. Finally, while we did not model resistance to second-line drugs, we assumed that such patients would become chronic cases and receive no curative benefit from treatment.

### Calibration

We used the model to simulate two pre-intervention stages, representing the natural history of untreated TB followed by a period of substandard treatment, in order to allocate the population to initial health states. During the substandard treatment stage, case finding and cure rates are low ( Table S2), and a relatively high proportion of treated cases acquire drug resistance and transmit MDR infection to susceptible persons (resulting in “primary” resistance). We varied combinations of model parameters so that the conditions at the end of the pre-intervention period were consistent with data from Peru describing both total incidence and the fraction of cases that are MDR-TB ( Table S3) [1, 17-20]. Since more than one combination of model parameters could generate an epidemic consistent with available data, several sets of parameter combinations were considered in sensitivity analysis ( Table S3).

For the base case, and the high and low MDR-TB fraction cases, we present in Table S4 alternative versions in which a different set of parameters is used to generate the conditions under consideration. For example, increasing the fraction of TB that is MDR-TB to 10% can be achieved by increasing the probability that resistance is acquired in treatment, or by assuming that the substandard treatment era is longer and has poorer treatment outcomes.

We found that an important feature of the calibration is the proportion of MDR-TB among previously treated patients re-entering treatment. This value varies by 5-10% over the course of the intervention, so we used the average value to compare to available data. A large study of laboratory results that were routinely collected for previously treated patients in Peru found that 57% were MDR-TB [21]. This study contrasts an earlier study based on a very small sample size that reported on 15.7% of previously-treated cases were MDR-TB. In our base case calibration, 53% of the persons entering the second round of treatment had MDR-TB. In general, however, varying the natural history parameters did not substantially affect the results provided the parameter sets were constrained to match calibration targets

### Simulation

Figure S1 shows the incidence trends for non-MDR TB and MDR-TB under the four non-dominated strategies (DOTS, STR2, ITR1, ITR2) in the base case. The horizontal axis marks time beginning with the introduction of pre-intervention treatment. During this phase, MDR-TB incidence rises. The incidence of non-MDR TB is the same for all strategies, since all strategies include first-line DOTS for non-MDR TB cases. Following initiation of the 30 year intervention phase, the incidence of MDR-TB declines under all strategies, but at different rates. Moreover, under the DOTS strategy, the decline in MDR-TB incidence reverses after a few years. Under DOTS-Plus strategies, the MDR-TB declines to a low equilibrium level for the duration of the intervention.

Figure S2 shows the five MDR-TB control strategies arranged in cost-effectiveness space. All programs except STR1 lie along the efficiency frontier. STR1 is dominated because it costs more than STR2 and provides no more benefit.

### Model Equations

The equations governing the transitions between model states are listed in Table S5.

1. Espinal MA, Simonsen L, Laszlo A, al. e (2000) Anti-tuberculosis drug resistance in the world, report no. 2, prevalence and trends. World Health Organization & International Union Against Tuberculosis and Lung Disease. (http://www.who.int/docstore/gtb/publications/drugresistance/PDF/fullversion.pdf)

2. Mitnick C, Bayona J, Palacios E, Shin S, Furin J, et al. (2003) Community-based therapy for multidrug-resistant tuberculosis in Lima, Peru. N Engl J Med 348: 119-128.

3. Tahaoglu K, Torun T, Sevim T (2001) The treatment of multidrug-resistant tuberculosis in Turkey. N Engl J Med 345: 170-174.

4. Suarez PG, Floyd K, Portocarrero J, Alarcon E, Rapiti E, et al. (2002) Feasibility and cost-effectiveness of standardised second-line drug treatment for chronic tuberculosis patients: a national cohort study in Peru. Lancet 359: 1980-1989.

5. Peru Ministry of Health (2001) Impacto Economico de la Tuberculosis en el Peru 1999. Tuberculosis en el Peru: Informe 2000. Lima.

6. Jacobs B, Clowes C, Wares F, Polivakho V, Lyagoshina T, et al. (2002) Cost-effectiveness analysis of the Russian treatment scheme for tuberculosis versus short-course chemotherapy: results from Tomsk, Siberia. Int J Tuberc Lung Dis 6: 396-405.

7. Murray CJL, DeJonghe E. (1991) Cost-effectiveness of chemotherapy for pulmonary tuberculosis in three sub-Saharan African countries. Lancet 338: 1305-1308.

8. Okello D, Floyd K, Adatu F, Odeke R, Gargioni G. (2003) Cost and cost-effectiveness of community-based care for tuberculosis patients in rural Uganda. Int J Tuberc Lung Dis 7:S72-S79.

9. Dye C, Scheele S, Dolin P, Pathania V, Raviglione MC (1999) Consensus statement. Global burden of tuberculosis: estimated incidence, prevalence, and mortality by country. WHO Global Surveillance and Monitoring Project. JAMA 282: 677-686.

10. Reider H (1999) Epidemiologic basis of tuberculosis control. Paris: International Union Against Tuberculosis and Lung Disease.

11. Behr MA, Warren SA, Salamon H, Hopewell PC, Ponce de Leon A, et al. (1999) Transmission of Mycobacterium tuberculosis from patients smear-negative for acid-fast bacilli. Lancet 353: 444-449.

12. Smith P MA (1994) Epidemiology of Tuberculosis. In: BR B, editor. Tuberculosis: Pathogenesis, Protection, and Control. Washington: ASM Press. pp. 47-59.

13. Comstock GW (1982) Epidemiology of tuberculosis. Am Rev Respir Dis 125: 8-15.

14. Vynnycky E, Fine PE (1997) The natural history of tuberculosis: the implications of age-dependent risks of disease and the role of reinfection. Epidemiol Infect 119: 183-201.

15. Global tuberculosis control: surveillance, planning, financing. WHO report 2005, World Health Organization ((WHO/HTM/TB/2005.349) (http://www.who.int/tb/publications/global_report/en/)

16. Dye C, Watt CJ, Bleed DM, Williams BG (2003) What is the limit to case detection under the DOTS strategy for tuberculosis control? Tuberculosis (Edinb) 83: 35-43.

17. Pablos-Mendez A RM, Laszlo A, et al. (1998) Global surveillance for antituberculosis-drug resistance 1994-1997. New Engl J Med 338: 1641-1649.

18. Styblo K (1986) Tuberculosis control and surveillance. In: Flenley DC PT, editor. Recent Advances in Respiratory Medicine. London: Churchill. pp. 77-108.

19. World Bank (2004) Peru at a glance. Accessed December 1, 2004 http://www.worldbank.org/data/countrydata/aag/per_aag.pdf

20. CIA (2005) The World Factbook. Accessed Jan 10, 2006. http://www.cia.gov/cia/publications/factbook/geos/pe.html

21. Vasquez-Campos L, Asencios-Solis L, Leo-Hurtado E, Quispe-Torres N, Salazar-Lindo E, et al. (2004) Drug resistance trends among previously treated tuberculosis patients in a national registry in Peru, 1994-2001. Int J Tuberc Lung Dis 8(4): 465-472.

Table S1. Itemized cost calculations for regimens using second line drugs * †

|  | **First line agents** | | **Injectable** | | **Fluoroquinolones** | | **Bacteriostatics** | | |
| --- | --- | --- | --- | --- | --- | --- | --- | --- | --- |
| **Standardized Regimen** | **E** | **Z** | **CM** | **KM** | **CPX** | **Moxi** | **Ethio** | **CS** | **PAS** |
| Form | tablets | tablets | vials | vials | tablets | tablets | tablets | capsules | sachets |
|  | 400mg | 500 mg | 1 gr | 1 gr | 500 mg | 400 mg | 250 mg | 250 mg | 4 gr |
| Duration of use (months) | 18 | 18 | 12 | 12 | 18 | 18 | 18 | 18 | 18 |
| Total quantity | 1404 | 1404 | 312 | 312 | 1404 | 468 | 1404 | 1404 | 936 |
| Percentage of patients | 100% | 100% | 25% | 75% | 95% | 5% | 100% | 100% | 100% |
| Cost per dose | $0.02 | $0.03 | $2.34 | $0.46 | $0.03 | $2.15 | $0.10 | $0.53 | $1.50 |
| Regimen cost per drug | $30 | $44 | $730 | $145 | $40 | $1,006 | $140 | $737 | $1404 |
|  |  |  |  |  |  |  |  |  |  |
| Total per patient cost: | Proportion of Patients |  |  |  |  |  |  |  |  |
| CPX, CS, E, Ethio, KM, Z, PAS | 0.7 | $2540 |  |  |  |  |  |  |  |
| CM, CPX, CS, E, Ethio, Z, PAS | 0.25 | $3126 |  |  |  |  |  |  |  |
| CS, E, Ethio, KM, Moxi, Z, PAS | 0.05 | $3507 |  |  |  |  |  |  |  |
| Avg. cost of drug for std. regimen |  | **$2735** |  |  |  |  |  |  |  |

*Table S1 continued*

|  | **Injectable** | | **Fluoroquinolones** | | **Bacteriostatics** | | |
| --- | --- | --- | --- | --- | --- | --- | --- |
| **Individualized Regimen**‡ | **CM** | **KM** | **CPX** | **Moxi** | **Ethio** | **CS** | **PAS** |
| Form | vials | vials | tablets | tablets | tablets | capsules | sachets |
|  | 1 gr | 1 gr | 500 mg | 400 mg | 250 mg | 250 mg | 4 gr |
| Duration of use (months) | 12 | 12 | 18 + | 18 + | 18 + | 18 + | 18 + |
| Total quantity (18 months) | 312 | 312 | 1404 | 468 | 1404 | 1404 | 936 |
| Total quantity (24 months) | 312 | 312 | 1872 | 624 | 1872 | 1872 | 1248 |
| Percentage of patients | 90% | 10% | 80% | 20% | 100% | 100% | 100% |
| Cost per dose | $2.34 | $0.46 | $0.03 | $2.15 | $0.10 | $0.53 | $1.50 |
| Regimen cost per drug (18 mo) | $730 | $145 | $40 | $1006 | $140 | $737 | $1404 |
| Regimen cost per drug (24 mo) | $730 | $145 | $53 | $1342 | $187 | $983 | $1872 |
|  |  |  |  |  |  |  |  |
| Total per patient cost: | Proportion of Patients |  |  |  |  |  |  |
| CM, CPX, CS, Ethio, PAS | 0.7 | $3051 |  |  |  |  |  |
| CM, CS, Ethio, Moxi, PAS | 0.2 | $4017 |  |  |  |  |  |
| CPX, CS, Ethio, KM, PAS | 0.1 | $2466 |  |  |  |  |  |
| Avg. cost of drug for 18 month |  | **$3186** |  |  |  |  |  |
| Avg cost of drug for 24 month |  | **$4024** |  |  |  |  |  |

* Source: unpublished information from Partners In Health. Injectables: Z = Streptomycin, CM = Capreomycin, KM = Kanamycin; Flouroquinolones: CPX = Ciprofloxacin, Moxi= Moxifloxacin; Bacteriostatic agents: CS = Cycloserine, E = Ethambutol, Ethoi= Ethionamide, PAS= Parra-aminosalicylic acid

†Drug prices reflect WHO Green Light Committee negotiated pricing as of August 2004.

‡24 month regimen used as based case.18 month individualized regimen presented as a sensitivity analysis.

Table S2. Treatment outcome parameters for substandard treatment era*

| New cases | | | | | | | | | | |
| --- | --- | --- | --- | --- | --- | --- | --- | --- | --- | --- |
|  | Non-MDR | | | | | MDR | | | | |
|  | %dead | %default | %cure | %fail |  | | %dead | % default | %cure | %fail |
| Substandard Treatment Era | 10 | 15 | 65 | 10 |  | | 12 | 32 | 35 | 21 |
| Previously treated cases | | | | | | | | | | |
|  | Non-MDR | | | | | MDR | | | | |
|  | %dead | %default | %cure | %fail |  | | %dead | % default | %cure | %fail |
| Substandard Treatment Era | 10 | 15 | 65 | 10 |  | | 12 | 32 | 35 | 21 |
| *The substandard treatment era was assumed to be 12 years long in the base case, with the annual case-finding increasing linearly from zero to 0.4 during the first 10 years and then remaining at 0.4. | | | | | | | | | | |

Table S3. Calibration to Peru and parameters used in sensitivity analysis

| Epidemiological Indicators | Peru Observed | Peru  Base Case | Peru  Base Case (Alt) | MDR-TB  Fitness = .5 | MDR Fraction = 10% | MDR Fraction = 10% (alt) | MDR Fraction = 1% | MDR Fraction = 1% (alt) | Double  TB burden |
| --- | --- | --- | --- | --- | --- | --- | --- | --- | --- |
| Annual TB Incidence (per 100,000) | 119 [9] | 118 | 119 | 119 | 122 | 118 | 116 | 119 | 238 |
| Proportion of prevalent TB that is MDR | 4.5% [1,17] | 4.6% | 4.6% | 4.6% | 9.7 | 9.9% | 1.2% | 1.0% | 4.5% |
| Proportion of new TB cases that are MDR | 2.4% [17] | 2.2% | 2.7% | 1.1% | 4.9% | 6.3% | 0.57 | 0.29% | 2.2% |
| Proportion of those entering a second round of treatment that have MDR-TB | 57%, [18 ] | 54% | 56% | 45% | 95% | 60% | 19% | 40% | 55% |
| Prevalence latent drug susceptible infection | 44% [9] | 56% | 47% | 56% | 56% | 56% | 54% | 56% | 74% |
| Duration untreated TB disease (years) | 2 [19] | 2 | 2 | 2 | 2 | 2 | 2 | 2 | 2 |
| Life expectancy (years) | 70 [20] | 67 | 67 | 67 | 67 | 67 | 67 | 67 | 67 |
| Annual population increase | 1.4% [21] | 1.4% | 1.4% | 1.4% | 1.4% | 1.4% | 1.4% | 1.4% | 1.4% |

( Table S3 continued)

|  | Peru  Base Case | Peru  Base Case (Alt) | MDR-TB  Fitness = .5 | MDR Fraction = 10% | MDR Fraction = 10% (Alt) | MDR Fraction = 1% | MDR Fraction = 1% (alt) | Double  TB burden |
| --- | --- | --- | --- | --- | --- | --- | --- | --- |
| Contacts per infectious case per year leading to smear-positive infection | 6.5 | 5.8 | 6.5 | 6.5 | 6.5 | 6.5 | 5.75 | 7.95 |
| Relative fitness of MDR strain for transmission | 1 | 1 | 0.5 | 1 | 1 | 1 | 1 | 1 |
| Annual probability of slow progression from latent to active disease | 0.0009 | 0.0009 | 0.0009 | 0.0009 | 0.0009 | .0009 | .0009 | 0.0013 |
| Proportion developing active disease soon after infection | 0.08 | 0.08 | 0.08 | 0.08 | 0.08 | 0.08 | 0.08 | 0.08 |
| Probability of acquiring MDR given treatment completion and failure | 0.4 | 0.4 | 0.4 | 0.9 | 0.4 | 0.1 | 0.4 | 0.4 |
| Annual case-finding rate | 0.19 | 0.056 | 0.195 | 0.19 | 0.19 | .19 | .06 | 0.17 |
| Substandard treatment stage duration (years) | 12 | 18 | 12 | 12 | 18.5 | 12 | 8 | 12 |
| Substandard treatment stage non-MDR cure rate | 0.65 | 0.45 | 0.57 | 0.65 | 0.52 | 0.65 | 0.65 | 0.65 |
| Substandard treatment stage MDR cure rate | 0.35 | 0.35 | 0.35 | 0.3 | 0.35 | 0.35 | 0.35 | 0.35 |

| Table S4. Sensitivity Analyses. (Extended version of Table 5 in the main text ) | | | | | | |
| --- | --- | --- | --- | --- | --- | --- |
|  | Strategy | Total Cost | Total QALY | Total TB Deaths | ICER: QALY | ICER: Averted Death |
|  | Base case |  |  |  |  |  |
|  | DOTS | $185,970 | 2,019,813 | 307.3 |  |  |
|  | STR1 | $272,340 | 2,019,872 | 302.5 | $1,467 | $17,871 |
|  | STR2 | $228,124 | 2,019,872 | 302.5 | $716 | $8,722 |
|  | ITR1 | $239,755 | 2,019,884 | 301.6 | $987 | $12,441 |
|  | ITR2 | $423,554 | 2,019,900 | 300.4 | $11,439 | $160,100 |
| 1 | Base case (undiscounted) | | |  |  |  |
|  | DOTS | $271,769 | 3,169,586 | 400.8 |  |  |
|  | STR1 | $397,846 | 3,169,693 | 393.4 | $1,178 | $16,901 |
|  | STR2 | $333,128 | 3,169,693 | 393.4 | $573 | $8,225 |
|  | ITR1 | $350,240 | 3,169,714 | 391.9 | $805 | $12,095 |
|  | ITR2 | $618,167 | 3,169,742 | 390.3 | $9,540 | $164,444 |
| 2 | Treatment does not reduce transmission (constant exogenous TB incidence rate) | | | | | |
|  | DOTS | $388,907 | 2,017,400 | 528.3 |  |  |
|  | STR1 | $582,805 | 2,017,461 | 522.6 | $3,172 | $33,937 |
|  | STR2 | $493,658 | 2,017,461 | 522.6 | $1,714 | $18,334 |
|  | ITR1 | $523,386 | 2,017,477 | 521.2 | $1,934 | $20,703 |
|  | ITR2 | $988,490 | 2,017,520 | 517.3 | $10,837 | $118,855 |
| 3 | Alternative Version of Base Case using different natural history parameters | | | | | |
|  | DOTS | $161,256 | 2,019,432 | 318.4 |  |  |
|  | STR1 | $238,020 | 2,019,483 | 314.3 | $1,522 | $18,564 |
|  | STR2 | $199,481 | 2,019,483 | 314.3 | $758 | $9,244 |
|  | ITR1 | $210,057 | 2,019,493 | 313.5 | $1,031 | $12,946 |
|  | ITR2 | $379,639 | 2,019,510 | 312.3 | $9,965 | $138,909 |
| 4 | First-line regimen costs $60 for new cases $100 for re-treatment | | | | | |
|  | DOTS | $31,987 | 2,019,813 | 307.3 |  |  |
|  | STR1 | $129,343 | 2,019,872 | 302.5 | $1,654 | $20,144 |
|  | STR2 | $80,127 | 2,019,872 | 302.5 | $818 | $9,961 |
|  | ITR1 | $92,005 | 2,019,884 | 301.6 | $1,008 | $12,704 |
|  | ITR2 | $279,944 | 2,019,900 | 300.4 | $11,696 | $163,707 |
| 5 | Effectiveness of first-line regimen versus MDR-TB reduced by 20% | | | | | |
|  | DOTS | $185,970 | 2,019,813 | 307.3 |  |  |
|  | STR1 | $275,974 | 2,019,848 | 304.6 | $2,580 | $32,445 |
|  | STR2 | $237,040 | 2,019,847 | 304.6 | $1,521 | $18,815 |
|  | ITR1 | $252,614 | 2,019,857 | 303.8 | $1,541 | $19,635 |
|  | ITR2 | $464,757 | 2,019,863 | 303.4 | $33,576 | $506,135 |
| 6 | Effectiveness of second-line regimens versus MDR-TB reduced by 20% | | | | | |
|  | DOTS | $189,926 | 2,019,756 | 312.4 |  |  |
|  | STR1 | $280,753 | 2,019,854 | 303.9 | $929 | $10,727 |
|  | STR2 | $236,567 | 2,019,854 | 303.9 | $477 | $5,509 |
|  | ITR1 | $249,202 | 2,019,868 | 302.7 | $880 | $10,883 |
|  | ITR2 | $423,554 | 2,019,900 | 300.4 | $5,541 | $75,603 |

|  | Strategy | Total Cost | Total QALY | Total TB Deaths | ICER: QALY | ICER: Averted Death |
| --- | --- | --- | --- | --- | --- | --- |
| 7 | Reduced relative fitness (transmissibility) of MDR-TB to 50% | | | | | |
|  | DOTS | $182,292 | 2,019,880 | 302.3 |  |  |
|  | STR1 | $267,203 | 2,019,919 | 299.3 | $2,160 | $28,199 |
|  | STR2 | $221,610 | 2,019,919 | 299.3 | $1,000 | $13,058 |
|  | ITR1 | $232,926 | 2,019,928 | 298.6 | $1,277 | $16,923 |
|  | ITR2 | $391,215 | 2,019,936 | 298.1 | $19,602 | $295,373 |
| 8 | Reduced relative fitness (transmissibility) of MDR-TB to 50% (Individual benefits only) | | | | | |
|  | DOTS | $383,507 | 2,017,461 | 522.7 |  |  |
|  | STR1 | $562,087 | 2,017,513 | 518.0 | $3,413 | $37,771 |
|  | STR2 | $470,494 | 2,017,513 | 518.0 | $1,663 | $18,399 |
|  | ITR1 | $495,518 | 2,017,526 | 516.8 | $1,902 | $21,062 |
|  | ITR2 | $873,778 | 2,017,549 | 514.8 | $16,633 | $188,151 |
| 9 | Reduced time-horizon to 10 years (QALYs from averted deaths that accrue beyond 10 years are ignored) | | | | | |
|  | DOTS | $95,483 | 788,537 | 218.5 |  |  |
|  | STR1 | $140,209 | 788,546 | 216.5 | $5,513 | $21,969 |
|  | STR2 | $117,629 | 788,546 | 216.5 | $2,730 | $10,878 |
|  | ITR1 | $123,528 | 788,547 | 216.0 | $3,422 | $13,762 |
|  | ITR2 | $219,263 | 788,551 | 215.4 | $28,907 | $145,544 |
| 10 | Reduced time-horizon to 10 years (Individual benefits only) | | | | |  |
|  | DOTS | $139,015 | 788,281 | 282.8 |  |  |
|  | STR1 | $203,372 | 788,287 | 281.1 | $10,798 | $37,833 |
|  | STR2 | $172,333 | 788,287 | 281.1 | $5,590 | $19,586 |
|  | ITR1 | $181,030 | 788,289 | 280.6 | $5,860 | $20,303 |
|  | ITR2 | $336,722 | 788,294 | 279.4 | $31,946 | $122,774 |
| 11 | MDR-TB represents 1% of all TB cases (probability of acquiring MDR-TB reduced) | | | | | |
|  | DOTS | $181,830 | 2,019,900 | 300.9 |  |  |
|  | STR1 | $253,467 | 2,019,917 | 299.6 | $4,201 | $53,826 |
|  | STR2 | $193,476 | 2,019,915 | 299.6 | $768 | $9,345 |
|  | ITR1 | $197,258 | 2,019,918 | 299.4 | $1,247 | $15,700 |
|  | ITR2 | $345,409 | 2,019,922 | 299.1 | $34,757 | $482,668 |
| 12 | MDR-TB represents 10% of all TB cases (probability of acquiring MDR-TB increased) | | | | | |
|  | DOTS | $192,575 | 2,019,660 | 318.2 |  |  |
|  | STR1 | $288,813 | 2,019,790 | 307.6 | $743 | $9,103 |
|  | STR2 | $283,499 | 2,019,790 | 307.6 | $703 | $8,609 |
|  | ITR1 | $307,707 | 2,019,816 | 305.6 | $934 | $11,838 |
|  | ITR2 | $545,062 | 2,019,850 | 303.2 | $6,937 | $98,924 |
| 13 | Case detection rate reduced to 50% per year | | | |  |  |
|  | DOTS | $176,787 | 2,018,546 | 400.0 |  |  |
|  | STR1 | $254,530 | 2,018,604 | 395.2 | $1,347 | $16,022 |
|  | STR2 | $215,011 | 2,018,604 | 395.2 | $662 | $7,878 |
|  | ITR1 | $225,533 | 2,018,615 | 394.2 | $923 | $11,349 |
|  | ITR2 | $401,431 | 2,018,631 | 393.1 | $11,227 | $153,716 |

|  | Strategy | Total Cost | Total QALY | Total TB Deaths | ICER: QALY | ICER: Averted Death |
| --- | --- | --- | --- | --- | --- | --- |
| 14 | Overall burden of smear positive TB doubled to 238 per 100,000 | | | | | |
|  | DOTS | $360,272 | 2,011,862 | 824.9 |  |  |
|  | STR1 | $520,650 | 2,011,992 | 813.3 | $1,237 | $13,753 |
|  | STR2 | $442,006 | 2,011,992 | 813.3 | $631 | $7,009 |
|  | ITR1 | $464,032 | 2,012,017 | 811.1 | $884 | $10,305 |
|  | ITR2 | $847,376 | 2,012,056 | 808.1 | $9,855 | $125,534 |
| 15 | Worst Case Scenario combining 4, 6, 7 & 9 | | | |  |  |
|  | DOTS | $16,267 | 788,550 | 215.9 |  |  |
|  | STR1 | $65,618 | 788,555 | 214.9 | $11,320 | $52,192 |
|  | STR2 | $42,913 | 788,554 | 215.0 | $6,772 | $30,505 |
|  | ITR1 | $50,219 | 788,555 | 214.7 | $6,442 | $28,866 |
|  | ITR2 | $139,820 | 788,556 | 214.5 | $91,431 | $571,776 |
| 16 | 10% MDR fraction (alternative calibration, increased failure rate & longer substandard treatment duration) | | | | | |
|  | DOTS | $187,516 | 2,019,888 | 303.9 |  |  |
|  | STR1 | $289,725 | 2,019,976 | 297.0 | $1,172 | $14,799 |
|  | STR2 | $246,414 | 2,019,976 | 297.0 | $675 | $8,528 |
|  | ITR1 | $262,448 | 2,019,993 | 295.7 | $922 | $12,048 |
|  | ITR2 | $506,499 | 2,020,031 | 293.0 | $6,411 | $92,036 |
| 17 | 1% MDR fraction (alt. calibration, lower force of infection, shorter substandard era with less case detection) | | | | | |
|  | DOTS | $158,197 | 2,019,412 | 318.3 |  |  |
|  | STR1 | $222,831 | 2,019,447 | 315.3 | $1,865 | $21,618 |
|  | STR2 | $186,477 | 2,019,447 | 315.3 | $816 | $9,459 |
|  | ITR1 | $194,364 | 2,019,454 | 314.7 | $1,114 | $13,292 |
|  | ITR2 | $330,149 | 2,019,459 | 314.3 | $27,278 | $336,097 |

Figure S1. Incidence trends.

Annual non-MDR and MDR-TB incidence per 100,000 population under the four non-dominated TB control strategies in the base case. MDR-TB is initially generated during the substandard treatment era which precedes the 30 year intervention era. MDR-TB incidence initially declines under all four control strategies, but when only first-line treatment is available, the decline reverses after a few years.

Figure S2. Efficiency frontier for MDR-TB control strategies.

In the base case, in order of increasing effectiveness and cost, DOTS, STR2, ITR1, ITR2 lie along the efficiency frontier. STR1 is dominated because it costs more than STR2 and provides no more benefit.

Table S5. Description of Model States

| **State Variables** | |
| --- | --- |
| Symbol | Description |
| $U$ | Number uninfected |
| $L_{d}$ | Number with latent non-MDR infection |
| $L_{m}$ | Number with latent MDR infections |
| $A1_{d}$ | Number with active untreated disease, non-MDR, never treated |
| $A2_{d}$ | Number with active untreated disease, non-MDR, previously treated |
| $A1_{m}$ | Number with active untreated disease, MDR, never treated |
| $A2_{m}$ | Number with active untreated disease, primary MDR, previously treated |
| $A3_{m}$ | Number with active untreated disease, acquired MDR, previously treated |
| $T1_{d}$ | Number with non-MDR disease, in first course of treatment |
| $T2_{d}$ | Number with non-MDR disease, in second course of treatment |
| $T1_{m}$ | Number with MDR disease, in first course of treatment |
| $T2_{m}$ | Number with primary MDR disease, in second course of treatment |
| $T3_{m}$ | Number with acquired MDR disease, in second course of treatment |
| $C_{d}$ | Number with chronic non-MDR disease |
| $C_{m}$ | Number with chronic MDR disease |
| $N$ | Total population $=U+L_{d}+L_{m}+A1_{d}+A2_{d}+A1_{m}+A2_{m}+A3_{m}+T1_{d}+T2_{d}+T1_{m}+T2_{m}+T3_{m}+C_{d}+C_{m}$ |

Table S6: Description of model parameters

| **Model parameters** | |
| --- | --- |
| $\pi$ | Number of persons entering the uninfected population per month |
| $\mu_{other}$ | Monthly death rate, no TB infection or latent infection |
| $\mu_{a}$ | Monthly death rate, untreated active TB |
| $\mu_{i}$ | Monthly death rate, treatment state i (i = $T1_{d}$,$T2_{d}$,$T1_{m}$,$T2_{m}$,$T3_{m}$) |
| $\delta_{i}$ | Monthly treatment default rate, treatment state i |
| $\xi_{i}$ | Monthly cure rate, treatment state i |
| $\tau_{i}$ | Monthly failure rate, treatment state i |
| $d_{i}$ | Fraction of treatment defaulters who have been cured before defaulting from treatment state i |
| $h_{i}$ | Fraction of persons leaving treatment state i uncured $=\frac{\delta_{i}(1-d_{i})+\tau_{i}+\mu_{i}}{\delta_{i}+\tau_{i}+\mu_{i}+\xi_{i}}$ |
| $\beta$ | Number of contacts per infectious case per month leading to infection |
| $f$ | Relative fitness of MDR strain for transmission |
| $\lambda_{d}$ | Monthly rate of new non-MDR infections $=\frac{\beta(A1_{d}+A2_{d}+T1_{d}h_{T1d}+T2_{d}h_{T2d})}{N}$ |
| $\lambda_{m}$ | Monthly rate of new primary MDR infections $=\frac{\beta f(A1_{m}+A2_{m}+A3_{m}+T1_{m}h_{T1m}+T2_{m}h_{T2m}+T3_{m}h_{T3m})}{N}$ |
| $q$ | Proportion developing active disease soon after infection |
| $1-v$ | Degree of partial immunity against reinfection if previously infected |
| $\rho$ | Monthly rate of spontaneous cure from untreated active disease |
| $\gamma$ | Monthly rate of progression from latent infection to active disease |
| $\varphi$ | Monthly rate of entering treatment from untreated active disease |
| $a$ | Probability of acquiring MDR given treatment completion and failure |
| $c$ | Ratio of probability of acquiring MDR given uncured default, relative to treatment failure |

**State Transition Equations**

Transitions between states are described by differential equations that reflect the net monthly rate of increase to the number of persons in each state. This rate of increase reflects flows of persons from and to each of the other states in the model. In the equations that follow, the monthly rate of increase in a state is denoted by a prime (‘), the symbol for the first (partial) derivative with respect to time. For example, $U'$ denotes the rate of increase in the number of uninfected persons, $L_{d}'$ denotes the rate of increase in the number of persons latently infected with non-MDR TB, etc.

$$U'=[\pi]-[\mu_{SL}+\lambda_{d}+\lambda_{m}]$$

$$L_{d}'=[U\lambda_{d}(1-q)+L_{m}\lambda_{d}(1-qv)+\rho(A1_{d}+A2_{d}+C_{d})+T1_{d}(\delta_{T1d}d_{T1d}+\xi_{T1d})+T2_{d}(\delta_{T2d}d_{T2d}+\xi_{T2d})]-[\mu_{SL}+\lambda_{d}qv+\lambda_{m}+\gamma]$$

$$\begin{matrix} L_{m}'=[U\lambda_{m}(1-q)+L_{d}\lambda_{m}(1-qv)+\rho(A1_{m}+A2_{m}+A3_{m}+C_{m})+T1_{m}(\delta_{T1m}d_{T1m}+\xi_{T1m})+T2_{m}(\delta_{T2m}d_{T2m}+\xi_{T2m})+T3_{m}(\delta_{T3m}d_{T3m}+\xi_{T3m})] \\ -[\mu_{SL}+\lambda_{m}qv+\lambda_{d}+\gamma] \end{matrix}$$

$$A1_{d}'=[U\lambda_{d}q+L_{d}\lambda_{d}qv+L_{m}\lambda_{d}qv+L_{d}\gamma]-[\mu_{a}+\varphi+\rho]$$

$$A1_{m}'=[U\lambda_{m}q+L_{m}\lambda_{m}qv+L_{d}\lambda_{m}qv+L_{m}\gamma]-[\mu_{a}+\varphi+\rho]$$

$$A2_{d}'=[T1_{d}\delta_{T1d}(1-d_{T1d})(1-ac)]-[\mu_{a}+\varphi+\rho]$$

$$A2_{m}'=[T1_{m}\delta_{T1m}(1-d_{T1m})]-[\mu_{a}+\varphi+\rho]$$

$$A3_{m}'=[T1_{d}\delta_{T1d}(1-d_{T1d})ac]-[\mu_{a}+\varphi+\rho]$$

$$T1_{d}'=[A1_{d}\varphi]-[\delta_{T1d}+\tau_{T1d}+\xi_{T1d}+\mu_{T1d}]$$

$$T1_{m}'=[A1_{m}\varphi]-[\delta_{T1m}+\tau_{T1m}+\xi_{T1m}+\mu_{T1m}]$$

$$T2_{d}'=[A2_{d}\varphi+T1_{d}\tau_{T1d}(1-a)]-[\delta_{T2d}+\tau_{T2d}+\xi_{T2d}+\mu_{T2d}]$$

$$T2_{m}'=[A2_{m}\varphi+T1_{m}\tau_{T1m}]-[\delta_{T2m}+\tau_{T2m}+\xi_{T2m}+\mu_{T2m}]$$

$$T3_{m}'=[A3_{m}\varphi+T1_{d}\tau_{T1d}a]-[\delta_{T3m}+\tau_{T3m}+\xi_{T3m}+\mu_{T3m}]$$

$$C_{d}'=[T2_{d}\delta_{T2d}(1-d_{T2d})(1-ac)+T2_{d}\tau_{T2d}(1-a)]-[\frac{\mu_{a}}{2}+\rho]$$

$$C_{m}'=[T2_{d}\delta_{T2d}(1-d_{T2d})ac+T2_{d}\tau_{T2d}a+T2_{m}\delta_{T2m}(1-d_{T2m})+T2_{m}\tau_{T2m}+T3_{m}\delta_{T3m}(1-d_{T3m})+T3_{m}\tau_{T3m}]-[\frac{\mu_{a}}{2}+\rho]$$
