# Supplementary material for: Cost-Effectiveness of Treating Multidrug-Resistant Tuberculosis
Source: PLoS Med. 2006 Jul 4;3(7):e241. doi: 10.1371/journal.pmed.0030241 (PMC1483913; doi:10.1371/journal.pmed.0030241)
Supplement: Supplementary file 2 [file pmed.0030241.st001.doc]

Table S1. Treatment outcome parameters for substandard treatment era*

| New cases | | | | | | | | | | |
| --- | --- | --- | --- | --- | --- | --- | --- | --- | --- | --- |
|  | Non-MDR | | | | | MDR | | | | |
|  | %dead | %default | %cure | %fail |  | | %dead | % default | %cure | %fail |
| Substandard Treatment Era | 10 | 15 | 65 | 10 |  | | 12 | 32 | 35 | 21 |
| Previously treated cases | | | | | | | | | | |
|  | Non-MDR | | | | | MDR | | | | |
|  | %dead | %default | %cure | %fail |  | | %dead | % default | %cure | %fail |
| Substandard Treatment Era | 10 | 15 | 65 | 10 |  | | 12 | 32 | 35 | 21 |
| *The substandard treatment era was assumed to be 12 years long in the base case, with the annual case-finding increasing linearly from zero to 0.4 during the first 10 years and then remaining at 0.4. | | | | | | | | | | |
