# Supplementary material for: Cost-Effectiveness of Treating Multidrug-Resistant Tuberculosis
Source: PLoS Med. 2006 Jul 4;3(7):e241. doi: 10.1371/journal.pmed.0030241 (PMC1483913; doi:10.1371/journal.pmed.0030241)
Supplement: Supplementary file 3 [file pmed.0030241.st002.doc]

Table S2. Description of Model States

| **State Variables** | |
| --- | --- |
| Symbol | Description |
| $U$ | Number uninfected |
| $L_{d}$ | Number with latent non-MDR infection |
| $L_{m}$ | Number with latent MDR infections |
| $A1_{d}$ | Number with active untreated disease, non-MDR, never treated |
| $A2_{d}$ | Number with active untreated disease, non-MDR, previously treated |
| $A1_{m}$ | Number with active untreated disease, MDR, never treated |
| $A2_{m}$ | Number with active untreated disease, primary MDR, previously treated |
| $A3_{m}$ | Number with active untreated disease, acquired MDR, previously treated |
| $T1_{d}$ | Number with non-MDR disease, in first course of treatment |
| $T2_{d}$ | Number with non-MDR disease, in second course of treatment |
| $T1_{m}$ | Number with MDR disease, in first course of treatment |
| $T2_{m}$ | Number with primary MDR disease, in second course of treatment |
| $T3_{m}$ | Number with acquired MDR disease, in second course of treatment |
| $C_{d}$ | Number with chronic non-MDR disease |
| $C_{m}$ | Number with chronic MDR disease |
| $N$ | Total population $=U+L_{d}+L_{m}+A1_{d}+A2_{d}+A1_{m}+A2_{m}+A3_{m}+T1_{d}+T2_{d}+T1_{m}+T2_{m}+T3_{m}+C_{d}+C_{m}$ |
