# Supplementary material for: Cost-Effectiveness of Treating Multidrug-Resistant Tuberculosis
Source: PLoS Med. 2006 Jul 4;3(7):e241. doi: 10.1371/journal.pmed.0030241 (PMC1483913; doi:10.1371/journal.pmed.0030241)
Supplement: Supplementary file 4 [file pmed.0030241.st003.doc]

Table S3: Description of model parameters

| **Model parameters** | |
| --- | --- |
| $\pi$ | Number of persons entering the uninfected population per month |
| $\mu_{other}$ | Monthly death rate, no TB infection or latent infection |
| $\mu_{a}$ | Monthly death rate, untreated active TB |
| $\mu_{i}$ | Monthly death rate, treatment state i (i = $T1_{d}$,$T2_{d}$,$T1_{m}$,$T2_{m}$,$T3_{m}$) |
| $\delta_{i}$ | Monthly treatment default rate, treatment state i |
| $\xi_{i}$ | Monthly cure rate, treatment state i |
| $\tau_{i}$ | Monthly failure rate, treatment state i |
| $d_{i}$ | Fraction of treatment defaulters who have been cured before defaulting from treatment state i |
| $h_{i}$ | Fraction of persons leaving treatment state i uncured $=\frac{\delta_{i}(1-d_{i})+\tau_{i}+\mu_{i}}{\delta_{i}+\tau_{i}+\mu_{i}+\xi_{i}}$ |
| $\beta$ | Number of contacts per infectious case per month leading to infection |
| $f$ | Relative fitness of MDR strain for transmission |
| $\lambda_{d}$ | Monthly rate of new non-MDR infections $=\frac{\beta(A1_{d}+A2_{d}+T1_{d}h_{T1d}+T2_{d}h_{T2d})}{N}$ |
| $\lambda_{m}$ | Monthly rate of new primary MDR infections $=\frac{\beta f(A1_{m}+A2_{m}+A3_{m}+T1_{m}h_{T1m}+T2_{m}h_{T2m}+T3_{m}h_{T3m})}{N}$ |
| $q$ | Proportion developing active disease soon after infection |
| $1-v$ | Degree of partial immunity against reinfection if previously infected |
| $\rho$ | Monthly rate of spontaneous cure from untreated active disease |
| $\gamma$ | Monthly rate of progression from latent infection to active disease |
| $\varphi$ | Monthly rate of entering treatment from untreated active disease |
| $a$ | Probability of acquiring MDR given treatment completion and failure |
| $c$ | Ratio of probability of acquiring MDR given uncured default, relative to treatment failure |

**State Transition Equations**

Transitions between states are described by differential equations that reflect the net monthly rate of increase to the number of persons in each state. This rate of increase reflects flows of persons from and to each of the other states in the model. In the equations that follow, the monthly rate of increase in a state is denoted by a prime (‘), the symbol for the first (partial) derivative with respect to time. For example, $U'$ denotes the rate of increase in the number of uninfected persons, $L_{d}'$ denotes the rate of increase in the number of persons latently infected with non-MDR TB, etc.

$$U'=[\pi]-[\mu_{SL}+\lambda_{d}+\lambda_{m}]$$

$$L_{d}'=[U\lambda_{d}(1-q)+L_{m}\lambda_{d}(1-qv)+\rho(A1_{d}+A2_{d}+C_{d})+T1_{d}(\delta_{T1d}d_{T1d}+\xi_{T1d})+T2_{d}(\delta_{T2d}d_{T2d}+\xi_{T2d})]-[\mu_{SL}+\lambda_{d}qv+\lambda_{m}+\gamma]$$

$$\begin{matrix} L_{m}'=[U\lambda_{m}(1-q)+L_{d}\lambda_{m}(1-qv)+\rho(A1_{m}+A2_{m}+A3_{m}+C_{m})+T1_{m}(\delta_{T1m}d_{T1m}+\xi_{T1m})+T2_{m}(\delta_{T2m}d_{T2m}+\xi_{T2m})+T3_{m}(\delta_{T3m}d_{T3m}+\xi_{T3m})] \\ -[\mu_{SL}+\lambda_{m}qv+\lambda_{d}+\gamma] \end{matrix}$$

$$A1_{d}'=[U\lambda_{d}q+L_{d}\lambda_{d}qv+L_{m}\lambda_{d}qv+L_{d}\gamma]-[\mu_{a}+\varphi+\rho]$$

$$A1_{m}'=[U\lambda_{m}q+L_{m}\lambda_{m}qv+L_{d}\lambda_{m}qv+L_{m}\gamma]-[\mu_{a}+\varphi+\rho]$$

$$A2_{d}'=[T1_{d}\delta_{T1d}(1-d_{T1d})(1-ac)]-[\mu_{a}+\varphi+\rho]$$

$$A2_{m}'=[T1_{m}\delta_{T1m}(1-d_{T1m})]-[\mu_{a}+\varphi+\rho]$$

$$A3_{m}'=[T1_{d}\delta_{T1d}(1-d_{T1d})ac]-[\mu_{a}+\varphi+\rho]$$

$$T1_{d}'=[A1_{d}\varphi]-[\delta_{T1d}+\tau_{T1d}+\xi_{T1d}+\mu_{T1d}]$$

$$T1_{m}'=[A1_{m}\varphi]-[\delta_{T1m}+\tau_{T1m}+\xi_{T1m}+\mu_{T1m}]$$

$$T2_{d}'=[A2_{d}\varphi+T1_{d}\tau_{T1d}(1-a)]-[\delta_{T2d}+\tau_{T2d}+\xi_{T2d}+\mu_{T2d}]$$

$$T2_{m}'=[A2_{m}\varphi+T1_{m}\tau_{T1m}]-[\delta_{T2m}+\tau_{T2m}+\xi_{T2m}+\mu_{T2m}]$$

$$T3_{m}'=[A3_{m}\varphi+T1_{d}\tau_{T1d}a]-[\delta_{T3m}+\tau_{T3m}+\xi_{T3m}+\mu_{T3m}]$$

$$C_{d}'=[T2_{d}\delta_{T2d}(1-d_{T2d})(1-ac)+T2_{d}\tau_{T2d}(1-a)]-[\frac{\mu_{a}}{2}+\rho]$$

$$C_{m}'=[T2_{d}\delta_{T2d}(1-d_{T2d})ac+T2_{d}\tau_{T2d}a+T2_{m}\delta_{T2m}(1-d_{T2m})+T2_{m}\tau_{T2m}+T3_{m}\delta_{T3m}(1-d_{T3m})+T3_{m}\tau_{T3m}]-[\frac{\mu_{a}}{2}+\rho]$$
