# Supplementary material for: Cost-Effectiveness of Treating Multidrug-Resistant Tuberculosis
Source: PLoS Med. 2006 Jul 4;3(7):e241. doi: 10.1371/journal.pmed.0030241 (PMC1483913; doi:10.1371/journal.pmed.0030241)
Supplement: Supplementary file 5 [file pmed.0030241.st004.doc]

Table S4. Calibration to Peru and parameters used in sensitivity analysis

| Epidemiological Indicators | Peru Observed | Peru  Base Case | Peru  Base Case (Alt) | MDR-TB  Fitness = .5 | MDR Fraction = 10% | MDR Fraction = 10% (alt) | MDR Fraction = 1% | MDR Fraction = 1% (alt) | Double  TB burden |
| --- | --- | --- | --- | --- | --- | --- | --- | --- | --- |
| Annual TB Incidence (per 100,000) | 119 [9] | 118 | 119 | 119 | 122 | 118 | 116 | 119 | 238 |
| Proportion of prevalent TB that is MDR | 4.5% [1,17] | 4.6% | 4.6% | 4.6% | 9.7 | 9.9% | 1.2% | 1.0% | 4.5% |
| Proportion of new TB cases that are MDR | 2.4% [17] | 2.2% | 2.7% | 1.1% | 4.9% | 6.3% | 0.57 | 0.29% | 2.2% |
| Proportion of those entering a second round of treatment that have MDR-TB | 57%, [18 ] | 54% | 56% | 45% | 95% | 60% | 19% | 40% | 55% |
| Prevalence latent drug susceptible infection | 44% [9] | 56% | 47% | 56% | 56% | 56% | 54% | 56% | 74% |
| Duration untreated TB disease (years) | 2 [19] | 2 | 2 | 2 | 2 | 2 | 2 | 2 | 2 |
| Life expectancy (years) | 70 [20] | 67 | 67 | 67 | 67 | 67 | 67 | 67 | 67 |
| Annual population increase | 1.4% [21] | 1.4% | 1.4% | 1.4% | 1.4% | 1.4% | 1.4% | 1.4% | 1.4% |

( Table S3 continued)

|  | Peru  Base Case | Peru  Base Case (Alt) | MDR-TB  Fitness = .5 | MDR Fraction = 10% | MDR Fraction = 10% (Alt) | MDR Fraction = 1% | MDR Fraction = 1% (alt) | Double  TB burden |
| --- | --- | --- | --- | --- | --- | --- | --- | --- |
| Contacts per infectious case per year leading to smear-positive infection | 6.5 | 5.8 | 6.5 | 6.5 | 6.5 | 6.5 | 5.75 | 7.95 |
| Relative fitness of MDR strain for transmission | 1 | 1 | 0.5 | 1 | 1 | 1 | 1 | 1 |
| Annual probability of slow progression from latent to active disease | 0.0009 | 0.0009 | 0.0009 | 0.0009 | 0.0009 | .0009 | .0009 | 0.0013 |
| Proportion developing active disease soon after infection | 0.08 | 0.08 | 0.08 | 0.08 | 0.08 | 0.08 | 0.08 | 0.08 |
| Probability of acquiring MDR given treatment completion and failure | 0.4 | 0.4 | 0.4 | 0.9 | 0.4 | 0.1 | 0.4 | 0.4 |
| Annual case-finding rate | 0.19 | 0.056 | 0.195 | 0.19 | 0.19 | .19 | .06 | 0.17 |
| Substandard treatment stage duration (years) | 12 | 18 | 12 | 12 | 18.5 | 12 | 8 | 12 |
| Substandard treatment stage non-MDR cure rate | 0.65 | 0.45 | 0.57 | 0.65 | 0.52 | 0.65 | 0.65 | 0.65 |
| Substandard treatment stage MDR cure rate | 0.35 | 0.35 | 0.35 | 0.3 | 0.35 | 0.35 | 0.35 | 0.35 |
