# Supplementary material for: Cost-Effectiveness of Treating Multidrug-Resistant Tuberculosis
Source: PLoS Med. 2006 Jul 4;3(7):e241. doi: 10.1371/journal.pmed.0030241 (PMC1483913; doi:10.1371/journal.pmed.0030241)
Supplement: Supplementary file 6 [file pmed.0030241.st005.doc]

Table S5. Itemized cost calculations for regimens using second line drugs * †

|  | **First line agents** | | **Injectable** | | **Fluoroquinolones** | | **Bacteriostatics** | | |
| --- | --- | --- | --- | --- | --- | --- | --- | --- | --- |
| **Standardized Regimen** | **E** | **Z** | **CM** | **KM** | **CPX** | **Moxi** | **Ethio** | **CS** | **PAS** |
| Form | tablets | tablets | vials | vials | tablets | tablets | tablets | capsules | sachets |
|  | 400mg | 500 mg | 1 gr | 1 gr | 500 mg | 400 mg | 250 mg | 250 mg | 4 gr |
| Duration of use (months) | 18 | 18 | 12 | 12 | 18 | 18 | 18 | 18 | 18 |
| Total quantity | 1404 | 1404 | 312 | 312 | 1404 | 468 | 1404 | 1404 | 936 |
| Percentage of patients | 100% | 100% | 25% | 75% | 95% | 5% | 100% | 100% | 100% |
| Cost per dose | $0.02 | $0.03 | $2.34 | $0.46 | $0.03 | $2.15 | $0.10 | $0.53 | $1.50 |
| Regimen cost per drug | $30 | $44 | $730 | $145 | $40 | $1,006 | $140 | $737 | $1404 |
|  |  |  |  |  |  |  |  |  |  |
| Total per patient cost: | Proportion of Patients |  |  |  |  |  |  |  |  |
| CPX, CS, E, Ethio, KM, Z, PAS | 0.7 | $2540 |  |  |  |  |  |  |  |
| CM, CPX, CS, E, Ethio, Z, PAS | 0.25 | $3126 |  |  |  |  |  |  |  |
| CS, E, Ethio, KM, Moxi, Z, PAS | 0.05 | $3507 |  |  |  |  |  |  |  |
| Avg. cost of drug for std. regimen |  | **$2735** |  |  |  |  |  |  |  |

*Table S1* continued

|  | **Injectable** | | **Fluoroquinolones** | | **Bacteriostatics** | | |
| --- | --- | --- | --- | --- | --- | --- | --- |
| **Individualized Regimen**‡ | **CM** | **KM** | **CPX** | **Moxi** | **Ethio** | **CS** | **PAS** |
| Form | vials | vials | tablets | tablets | tablets | capsules | sachets |
|  | 1 gr | 1 gr | 500 mg | 400 mg | 250 mg | 250 mg | 4 gr |
| Duration of use (months) | 12 | 12 | 18 + | 18 + | 18 + | 18 + | 18 + |
| Total quantity (18 months) | 312 | 312 | 1404 | 468 | 1404 | 1404 | 936 |
| Total quantity (24 months) | 312 | 312 | 1872 | 624 | 1872 | 1872 | 1248 |
| Percentage of patients | 90% | 10% | 80% | 20% | 100% | 100% | 100% |
| Cost per dose | $2.34 | $0.46 | $0.03 | $2.15 | $0.10 | $0.53 | $1.50 |
| Regimen cost per drug (18 mo) | $730 | $145 | $40 | $1006 | $140 | $737 | $1404 |
| Regimen cost per drug (24 mo) | $730 | $145 | $53 | $1342 | $187 | $983 | $1872 |
|  |  |  |  |  |  |  |  |
| Total per patient cost: | Proportion of Patients |  |  |  |  |  |  |
| CM, CPX, CS, Ethio, PAS | 0.7 | $3051 |  |  |  |  |  |
| CM, CS, Ethio, Moxi, PAS | 0.2 | $4017 |  |  |  |  |  |
| CPX, CS, Ethio, KM, PAS | 0.1 | $2466 |  |  |  |  |  |
| Avg. cost of drug for 18 month |  | **$3186** |  |  |  |  |  |
| Avg cost of drug for 24 month |  | **$4024** |  |  |  |  |  |

* Source: unpublished information from Partners In Health. Injectables: Z = Streptomycin, CM = Capreomycin, KM = Kanamycin; Flouroquinolones: CPX = Ciprofloxacin, Moxi= Moxifloxacin; Bacteriostatic agents: CS = Cycloserine, E = Ethambutol, Ethoi= Ethionamide, PAS= Parra-aminosalicylic acid

†Drug prices reflect WHO Green Light Committee negotiated pricing as of August 2004.

‡24 month regimen used as based case.18 month individualized regimen presented as a sensitivity analysis.
