# Supplementary material for: Cost-Effectiveness of Treating Multidrug-Resistant Tuberculosis
Source: PLoS Med. 2006 Jul 4;3(7):e241. doi: 10.1371/journal.pmed.0030241 (PMC1483913; doi:10.1371/journal.pmed.0030241)
Supplement: Supplementary file 7 [file pmed.0030241.st006.doc]

| Table S6. Sensitivity Analyses. (Extended version of Table 5 in the main text ) | | | | | | |
| --- | --- | --- | --- | --- | --- | --- |
|  | Strategy | Total Cost | Total QALY | Total TB Deaths | ICER: QALY | ICER: Averted Death |
|  | Base case |  |  |  |  |  |
|  | DOTS | $185,970 | 2,019,813 | 307.3 |  |  |
|  | STR1 | $272,340 | 2,019,872 | 302.5 | $1,467 | $17,871 |
|  | STR2 | $228,124 | 2,019,872 | 302.5 | $716 | $8,722 |
|  | ITR1 | $239,755 | 2,019,884 | 301.6 | $987 | $12,441 |
|  | ITR2 | $423,554 | 2,019,900 | 300.4 | $11,439 | $160,100 |
| 1 | Base case (undiscounted) | | |  |  |  |
|  | DOTS | $271,769 | 3,169,586 | 400.8 |  |  |
|  | STR1 | $397,846 | 3,169,693 | 393.4 | $1,178 | $16,901 |
|  | STR2 | $333,128 | 3,169,693 | 393.4 | $573 | $8,225 |
|  | ITR1 | $350,240 | 3,169,714 | 391.9 | $805 | $12,095 |
|  | ITR2 | $618,167 | 3,169,742 | 390.3 | $9,540 | $164,444 |
| 2 | Treatment does not reduce transmission (constant exogenous TB incidence rate) | | | | | |
|  | DOTS | $388,907 | 2,017,400 | 528.3 |  |  |
|  | STR1 | $582,805 | 2,017,461 | 522.6 | $3,172 | $33,937 |
|  | STR2 | $493,658 | 2,017,461 | 522.6 | $1,714 | $18,334 |
|  | ITR1 | $523,386 | 2,017,477 | 521.2 | $1,934 | $20,703 |
|  | ITR2 | $988,490 | 2,017,520 | 517.3 | $10,837 | $118,855 |
| 3 | Alternative Version of Base Case using different natural history parameters | | | | | |
|  | DOTS | $161,256 | 2,019,432 | 318.4 |  |  |
|  | STR1 | $238,020 | 2,019,483 | 314.3 | $1,522 | $18,564 |
|  | STR2 | $199,481 | 2,019,483 | 314.3 | $758 | $9,244 |
|  | ITR1 | $210,057 | 2,019,493 | 313.5 | $1,031 | $12,946 |
|  | ITR2 | $379,639 | 2,019,510 | 312.3 | $9,965 | $138,909 |
| 4 | First-line regimen costs $60 for new cases $100 for re-treatment | | | | | |
|  | DOTS | $31,987 | 2,019,813 | 307.3 |  |  |
|  | STR1 | $129,343 | 2,019,872 | 302.5 | $1,654 | $20,144 |
|  | STR2 | $80,127 | 2,019,872 | 302.5 | $818 | $9,961 |
|  | ITR1 | $92,005 | 2,019,884 | 301.6 | $1,008 | $12,704 |
|  | ITR2 | $279,944 | 2,019,900 | 300.4 | $11,696 | $163,707 |
| 5 | Effectiveness of first-line regimen versus MDR-TB reduced by 20% | | | | | |
|  | DOTS | $185,970 | 2,019,813 | 307.3 |  |  |
|  | STR1 | $275,974 | 2,019,848 | 304.6 | $2,580 | $32,445 |
|  | STR2 | $237,040 | 2,019,847 | 304.6 | $1,521 | $18,815 |
|  | ITR1 | $252,614 | 2,019,857 | 303.8 | $1,541 | $19,635 |
|  | ITR2 | $464,757 | 2,019,863 | 303.4 | $33,576 | $506,135 |
| 6 | Effectiveness of second-line regimens versus MDR-TB reduced by 20% | | | | | |
|  | DOTS | $189,926 | 2,019,756 | 312.4 |  |  |
|  | STR1 | $280,753 | 2,019,854 | 303.9 | $929 | $10,727 |
|  | STR2 | $236,567 | 2,019,854 | 303.9 | $477 | $5,509 |
|  | ITR1 | $249,202 | 2,019,868 | 302.7 | $880 | $10,883 |
|  | ITR2 | $423,554 | 2,019,900 | 300.4 | $5,541 | $75,603 |

|  | Strategy | Total Cost | Total QALY | Total TB Deaths | ICER: QALY | ICER: Averted Death |
| --- | --- | --- | --- | --- | --- | --- |
| 7 | Reduced relative fitness (transmissibility) of MDR-TB to 50% | | | | | |
|  | DOTS | $182,292 | 2,019,880 | 302.3 |  |  |
|  | STR1 | $267,203 | 2,019,919 | 299.3 | $2,160 | $28,199 |
|  | STR2 | $221,610 | 2,019,919 | 299.3 | $1,000 | $13,058 |
|  | ITR1 | $232,926 | 2,019,928 | 298.6 | $1,277 | $16,923 |
|  | ITR2 | $391,215 | 2,019,936 | 298.1 | $19,602 | $295,373 |
| 8 | Reduced relative fitness (transmissibility) of MDR-TB to 50% (Individual benefits only) | | | | | |
|  | DOTS | $383,507 | 2,017,461 | 522.7 |  |  |
|  | STR1 | $562,087 | 2,017,513 | 518.0 | $3,413 | $37,771 |
|  | STR2 | $470,494 | 2,017,513 | 518.0 | $1,663 | $18,399 |
|  | ITR1 | $495,518 | 2,017,526 | 516.8 | $1,902 | $21,062 |
|  | ITR2 | $873,778 | 2,017,549 | 514.8 | $16,633 | $188,151 |
| 9 | Reduced time-horizon to 10 years (QALYs from averted deaths that accrue beyond 10 years are ignored) | | | | | |
|  | DOTS | $95,483 | 788,537 | 218.5 |  |  |
|  | STR1 | $140,209 | 788,546 | 216.5 | $5,513 | $21,969 |
|  | STR2 | $117,629 | 788,546 | 216.5 | $2,730 | $10,878 |
|  | ITR1 | $123,528 | 788,547 | 216.0 | $3,422 | $13,762 |
|  | ITR2 | $219,263 | 788,551 | 215.4 | $28,907 | $145,544 |
| 10 | Reduced time-horizon to 10 years (Individual benefits only) | | | | |  |
|  | DOTS | $139,015 | 788,281 | 282.8 |  |  |
|  | STR1 | $203,372 | 788,287 | 281.1 | $10,798 | $37,833 |
|  | STR2 | $172,333 | 788,287 | 281.1 | $5,590 | $19,586 |
|  | ITR1 | $181,030 | 788,289 | 280.6 | $5,860 | $20,303 |
|  | ITR2 | $336,722 | 788,294 | 279.4 | $31,946 | $122,774 |
| 11 | MDR-TB represents 1% of all TB cases (probability of acquiring MDR-TB reduced) | | | | | |
|  | DOTS | $181,830 | 2,019,900 | 300.9 |  |  |
|  | STR1 | $253,467 | 2,019,917 | 299.6 | $4,201 | $53,826 |
|  | STR2 | $193,476 | 2,019,915 | 299.6 | $768 | $9,345 |
|  | ITR1 | $197,258 | 2,019,918 | 299.4 | $1,247 | $15,700 |
|  | ITR2 | $345,409 | 2,019,922 | 299.1 | $34,757 | $482,668 |
| 12 | MDR-TB represents 10% of all TB cases (probability of acquiring MDR-TB increased) | | | | | |
|  | DOTS | $192,575 | 2,019,660 | 318.2 |  |  |
|  | STR1 | $288,813 | 2,019,790 | 307.6 | $743 | $9,103 |
|  | STR2 | $283,499 | 2,019,790 | 307.6 | $703 | $8,609 |
|  | ITR1 | $307,707 | 2,019,816 | 305.6 | $934 | $11,838 |
|  | ITR2 | $545,062 | 2,019,850 | 303.2 | $6,937 | $98,924 |
| 13 | Case detection rate reduced to 50% per year | | | |  |  |
|  | DOTS | $176,787 | 2,018,546 | 400.0 |  |  |
|  | STR1 | $254,530 | 2,018,604 | 395.2 | $1,347 | $16,022 |
|  | STR2 | $215,011 | 2,018,604 | 395.2 | $662 | $7,878 |
|  | ITR1 | $225,533 | 2,018,615 | 394.2 | $923 | $11,349 |
|  | ITR2 | $401,431 | 2,018,631 | 393.1 | $11,227 | $153,716 |

|  | Strategy | Total Cost | Total QALY | Total TB Deaths | ICER: QALY | ICER: Averted Death |
| --- | --- | --- | --- | --- | --- | --- |
| 14 | Overall burden of smear positive TB doubled to 238 per 100,000 | | | | | |
|  | DOTS | $360,272 | 2,011,862 | 824.9 |  |  |
|  | STR1 | $520,650 | 2,011,992 | 813.3 | $1,237 | $13,753 |
|  | STR2 | $442,006 | 2,011,992 | 813.3 | $631 | $7,009 |
|  | ITR1 | $464,032 | 2,012,017 | 811.1 | $884 | $10,305 |
|  | ITR2 | $847,376 | 2,012,056 | 808.1 | $9,855 | $125,534 |
| 15 | Worst Case Scenario combining 4, 6, 7 & 9 | | | |  |  |
|  | DOTS | $16,267 | 788,550 | 215.9 |  |  |
|  | STR1 | $65,618 | 788,555 | 214.9 | $11,320 | $52,192 |
|  | STR2 | $42,913 | 788,554 | 215.0 | $6,772 | $30,505 |
|  | ITR1 | $50,219 | 788,555 | 214.7 | $6,442 | $28,866 |
|  | ITR2 | $139,820 | 788,556 | 214.5 | $91,431 | $571,776 |
| 16 | 10% MDR fraction (alternative calibration, increased failure rate & longer substandard treatment duration) | | | | | |
|  | DOTS | $187,516 | 2,019,888 | 303.9 |  |  |
|  | STR1 | $289,725 | 2,019,976 | 297.0 | $1,172 | $14,799 |
|  | STR2 | $246,414 | 2,019,976 | 297.0 | $675 | $8,528 |
|  | ITR1 | $262,448 | 2,019,993 | 295.7 | $922 | $12,048 |
|  | ITR2 | $506,499 | 2,020,031 | 293.0 | $6,411 | $92,036 |
| 17 | 1% MDR fraction (alt. calibration, lower force of infection, shorter substandard era with less case detection) | | | | | |
|  | DOTS | $158,197 | 2,019,412 | 318.3 |  |  |
|  | STR1 | $222,831 | 2,019,447 | 315.3 | $1,865 | $21,618 |
|  | STR2 | $186,477 | 2,019,447 | 315.3 | $816 | $9,459 |
|  | ITR1 | $194,364 | 2,019,454 | 314.7 | $1,114 | $13,292 |
|  | ITR2 | $330,149 | 2,019,459 | 314.3 | $27,278 | $336,097 |
